# Weighting scheme for robust depth normalization in reflectance time-domain diffuse optical tomography

**DOI:** 10.1117/1.JBO.31.9.097002

**Published:** 2026-09-15

**Authors:** Ifechi A. E. Ejidike, Michael G. Tanner

**Affiliations:** Heriot-Watt University, Institute of Photonics and Quantum Sciences, Edinburgh, United Kingdom

**Keywords:** diffuse optics, diffuse optical imaging, diffuse optical tomography, finite element method, algorithm, reconstruction

## Abstract

**Significance:**

Reflectance mode diffuse optical tomography (DOT) is limited in its depth sensitivity. Current approaches to improve depth sensitivity need some prior knowledge to correctly localize deep inclusions. It has been shown that time-domain (TD) measurements can enhance depth sensitivity, but reconstructions still tend to be biased toward the surface, and TD modeling of diffuse light is computationally burdensome. Efficient TD modeling of diffuse light and prior-free reflectance DOT is critical to streamline the adoption of the technology into clinics.

**Aim:**

Our aim is to develop an efficient simulation tool for TD fluence in tissue as well a weighted reconstruction algorithm that uses TD measurements to enhance both the depth sensitivity and spatial selectivity of DOT without relying on any prior knowledge of the tissue structure or inclusion location.

**Approach:**

To overcome the computational burden of TD modeling of diffuse light, we solve the diffusion approximation in the Fourier domain for many frequencies parallelizing our solver across mesh nodes, sources, and frequencies. Using only the sensitivity matrix calculated from this modeled fluence, we calculate a weighting matrix that optimizes the spatial selectivity and depth sensitivity of our measurements. This weighting matrix was used in a Levenburg–Marquardt algorithm to retrieve the three-dimensional absorption profile of single inclusions at different depths, two complex scenes with inclusion(s) that spanned the depth of the sample, and computational phantoms adapted from publicly available breast lesion magnetic resonance imaging data.

**Results:**

We found in our simulations that our weighted scheme enables enhanced spatial selectivity for diffuse measurements as well as flattening the net sensitivity across the depth of the sample for DOT. We were able to accurately localize inclusions up to 40 mm deep without using prior knowledge of inclusion depth to normalize the sensitivity. Investigations of the effect of shot noise and Monte-Carlo-generated data on our reconstructions revealed we are still able to localize inclusions up to 30 mm deep for realistic noise levels. The complex targets were also well reconstructed up to 40 mm for noise-free measurements and up to 30 mm for the same noise levels. We found our regularization parameters had limited dependence on the geometry or ground truth optical property distribution. For all our reconstructions, we only tuned the regularizer used to find the weighting matrix based on the noise level.

**Conclusions:**

We demonstrate by simulation a weighting scheme for diffuse measurements and DOT that greatly enhances spatial selectivity and depth sensitivity, with no explicit depth prior. As well as this, we demonstrate the robustness of our approach by reconstructing complex targets with the same initial parameters used throughout.

## 1 Introduction

Diffuse optical tomography (DOT) is a macroscopic near-infrared (NIR) imaging technique that reconstructs a three-dimensional (3D) distribution of the absorption and scattering coefficients of tissue based on multiple measurements on the surface from known source locations.^1^^,^^2^ These optical properties can be mapped to physiological properties^3^ such as blood oxygenation and cell density or can be used to identify tumorous lesions.^4^ It is an optical technique that does not rely on ionizing radiation such as X-ray computed tomography (CT) and produces volumetric images like magnetic resonance imaging (MRI) without hazardous magnetic fields. Typically, to find the optical property distribution, the finite element method (FEM) is used to model the fluence within the tissue and at the boundaries;^5^ by comparing these boundary measurements to the modeled data, we can iteratively reconstruct the internal optical properties. In software such as NIRFAST^6^ and TOAST++,^7^ the FEM solution of the diffusion approximation is solved, and then the optical properties are updated such that the modeled data match the measured data. Some more computationally demanding methods that rely on solving the radiative transfer equation^8^ or mesh-based Monte Carlo modeling^9^^,^^10^ have been demonstrated, but generally, in human tissue, the diffusion approximation is valid.

DOT has been shown to identify cancerous lesions in breast tissue with good efficacy.^11^^,^^12^ Compared with CT and MRI, the resolution of DOT is very limited; this has been curtailed with various multimodal approaches that use structural information from CT, MRI, or ultrasound images to derive priors to guide the reconstruction process and enhance resolution.^13^^–^^15^ The instrumentation of multimodal systems and transmission-based DOT is a significant barrier of entry for clinical adoption when the selectivity and sensitivity of CT and MRI are already so high and are much more familiar to clinicians. DOT has demonstrated itself as a viable option in low-resource settings by leveraging the compact form factor available to it as an optical method,^16^^–^^19^ but this requires sensing to be done in the reflectance geometry. Diffuse optical techniques in this geometry have been shown to be effective for quantitative monitoring of breast cancer treatment,^20^^–^^22^ but DOT in reflectance geometry has a very limited depth sensitivity. Patient outcome for breast cancer is more positive when diagnosis is early, and we feel that enhancing the depth sensitivity of reflectance methods would place DOT as an effective early cancer detection method in community or low resource settings.

It has been shown that time-domain (TD)-DOT measurements that use pulsed laser sources can enhance sensitivity at depth compared with frequency-domain (FD) or continuous wave measurements.^23^^,^^24^ Photomultiplier tube TD detection schemes require bulky external hardware.^11^^,^^12^^,^^25^ Silicon photomultipliers have shown their efficacy as a compact solution to TD-DOT detection^26^^,^^27^ but still require external time-tagging electronics. Silicon single-photon avalanche diode (SPAD) arrays offer themselves as an excellent detector for TD-DOT due to their high quantum efficiency and compact, monolithic design with time tagging integrated directly on chip that allows for independent pixel read-out. This allows for multichannel measurements on a single detector array similar to intensified charge-coupled device cameras^28^^–^^30^ but with much finer timing resolution and better performance in low-light regimes. SPADs have been previously demonstrated as an avenue to compact reflectance measurements for TD-DOT.^24^^,^^31^^,^^32^ The system described by Jiang et al.^31^^–^^33^ is even compact enough to be handheld and due to the use of SPAD array is extremely high density with more than 2000 channels.

Solving the time-domain diffusion approximation is computationally taxing which warrants the use of TD moment data types which can be solved for directly,^34^ or the measured distributed time of flight (DTOF) is Fourier-transformed and the amplitude and phase of the most significant component are compared with FD solutions of the diffusion approximation. This means that the depth sensitivity of these systems is limited by the source–detector separation (SDS) of the optodes, and in the reflectance geometry, reconstructions are strongly biased toward the surface where the optodes lie. So, it is common practice to weight the reconstructions based on depth;^27^^,^^35^^–^^37^ this requires some prior knowledge of the target to pick the correct weightings, and if these weightings are picked incorrectly, it biases the reconstruction to the wrong depth.

In this work, we present a method for fast TD-DOT reconstructions based on time-gated data types as described by Orive-Miguel et al.^38^ These gated measurements have a sensitivity that is dependent on both the SDS and the timing. Later gates are sensitive to deeper regions of the sample, whereas earlier gates are not. We solve the diffusion equation by decomposing it to a truncated Fourier series^39^^,^^40^ and calculate the time-gated data types and sensitivities directly from this, completely in parallel. Enabled by the temporal selectivity of TD-gated data types, we demonstrate by simulation a weighting scheme that is derived directly from the sensitivity profiles that can be used to enhance the depth sensitivity and spatial selectivity of TD diffuse measurements. This weighting is used in a Levenburg–Marquardt (LM) algorithm to reconstruct the absorption profile of simulated samples and to simultaneously reconstruct the absorption and scattering profiles of segmented breast MRI data. Like other schemes, it does introduce another parameter to tune, but as we show, this is only tuned based on measurement noise. We calculate our weightings to flatten the net sensitivity across the depth of our simulated target, and as a result, the regularization parameters are not coupled as strongly to the location or shape of the inclusion, resulting in a weighting scheme that does not rely on prior structural information.

## 2 Theory

### 2.1 Fourier Truncation of the Diffusion Approximation

NIR fluence within tissue can be described by the diffusion approximation (DA) which is a special solution of the radiative transport equation where the absorption coefficient $\mu_{a}$ is much less than the reduced scattering coefficient $\mu_{s}^{\prime }$.^41^^,^^42^ In the frequency domain, this is given as

$$\frac{i\omega }{c}\phi (r,\omega )-\nabla \cdot \kappa (r)\nabla \phi (r,\omega )+\mu_{a}(r)\phi (r,\omega )=q(r,\omega ),$$ (1)

where ϕ(r,ω) is the photon fluence rate, c is the speed of light in the medium, q(r,ω) is an isotropic source term, and κ(r) is the diffusion coefficient defined as

$$\kappa (r)=\frac{1}{3[\mu_{a}(r)+\mu_{s}^{\prime }(r)]}.$$ (2)

In the FEM representation of the DA, we discretize our domain (i.e., the tissue) to the NN nodes in a tetrahedral mesh and finding the fluence amounts to solving the linear system^5^^,^^6^

A(ω)ϕ(ω)=Q(ω), (3)

where $A(\omega )\in C^{NN\times NN}$ is some transform that encodes the optical properties of the modeled mesh as well as the boundary conditions, and $\phi (\omega )\in C^{NN\times 1}$ and $Q(\omega )\in C^{NN\times 1}$ are the fluence and source defined on the mesh, respectively.

As described by Mozumder and Tarvainen,^39^^,^^40^ we decompose our source term into its truncated Fourier series coefficients such that

$$q(\omega_{k})=\frac{1}{T}\int_{0}^{T}q(t)e^{-i\omega_{k}t}.$$ (4)

We can then solve for the Fourier coefficients of the fluence and use Parseval’s theorem to find the time-gated fluence ϕ(G), where $G(\omega_{k})$ is the k’th Fourier coefficient of a time gate G(t):^38^

$$\overset{k}{\underset{n=-k}{\sum }}\phi (\omega_{k})\overset{¯}{G(\omega_{k})}\approx \frac{1}{T}\int_{0}^{T}\phi (t)G(t)=\phi (G).$$ (5)

Such that $\phi (G)\in R^{NN\times 1}$. Solving for the time-gated fluence this way has the advantage of not requiring finite time differencing, and given that the fluence is temporally diffuse, most of the signal lies in the lower frequencies. From the fluence, we define our boundary measurements used in the reconstruction as $\phi^{C}\in R^{NM\times 1}$, where NM is the number of measurements.

### 2.2 Weighted Reconstruction

The core of the DOT problem is internal optical property recovery from boundary measurements; this is done by minimizing the 2-norm of the residual between measurements $\phi^{M}$ and modeled data $\phi^{C}$ with respect to the optical properties. We can write the objective function as

$$\Omega =\{\delta \phi^{T}\delta \phi \},$$ (6)

where $\delta \phi =\phi^{M}-\phi^{C}\in R^{NM\times 1}$. To minimize Eq. (6), we use an iterative LM algorithm^43^^–^^45^ with some regularization λ to stabilize the inversion of the Hessian matrix $J^{T}J$,

$$[J^{T}J+\lambda I]\Delta \mu_{i}=J^{T}\delta \phi_{i-1},$$ (7)

where at each iteration i, the optical property update $\Delta \mu_{i}\in R^{NP\times 1}$ is solved for until the residual is minimized. NP is the number of optical properties to reconstruct. This can be absorption at every node, the reduced scattering, chromophore concentration, etc. The matrix $J\in R^{NM\times NP}$ is the sensitivity matrix defined as $\frac{\partial \phi^{C}}{\partial \mu }$, i.e., it describes the measured fluence response to a change in optical properties. We approximate the TD-gated sensitivity using Eq. (5).

Given that our residual vector δϕ is populated by different gated fluences at different surface locations, we have a large sensitivity overlap near the surface as well as some depth selectivity enabled by the gating. This bias toward the surface in our sensitivity profile is why weighting is needed to enhance the depth sensitivity. Typically, these weights are defined as dependent on node depth,^36^^–^^38^ but weighting such as this has two problems. First, scaling the sensitivity matrix on the right-hand side like this results in a solution that does not minimize the original objective function, so additional steps must be taken to properly rescale the minimized solution. Second, it just shifts the reconstructed optical properties deeper into the sample; in the case of a single inclusion, this requires some prior knowledge of the inclusion depth. For scenes where you have a mixture of shallow and deep inclusions, more complex normalization is needed as sensitivity is a function of node position in 3D, not just node depth.

LM minimization of Eq. (6) requires us to ignore higher-order terms of the residual such that

JΔμ=δϕ. (8)

We can analogize this as a more basic imaging problem, where J is our illumination pattern, Δμ is the target, and δϕ is the image we form. A similar problem is seen when trying to image through weakly scattering media such as multimode optical fibers, where the light input to the proximal facet is scrambled at the distal facet resulting in illumination patterns ill-suited for imaging. One method to produce patterns well-suited for imaging is to characterize the transmission matrix of the fiber such that the various input modes at the proximal facet can be selectively excited to form orthogonal scanning spots at the distal facet.^46^^–^^48^ Rather than selective excitation, we can use the sensitivity matrix in a similar way to find a weighted linear combination of each residual that forms more favorable sensitivities, which are not as sensitive to the surface and have low spatial correlation with each other. For a target sensitivity $J_{s}$, we find the weighting matrix w that forms this sensitivity by solving

$$wJ=J_{s},$$ (9)

where $w\in R^{NJ\times NM}$ and $J_{s}\in R^{NJ\times NN}$. This distribution can be a single localized sensitivity to look at one region in the sample (NJ=1) or an array of sensitivities that may be used to form a reconstruction basis (NJ>1). As J is, in general, a fat matrix, we stabilize the inversion with $\lambda_{s}$

$$w(JJ^{T}+\lambda_{s}I)=J_{s}J^{T}.$$ (10)

With our previous assertion that the measurement residual and sensitivity are linearly related, it must be the case that

$$\frac{\partial w\phi }{\partial \mu }=\mathrm{wJ}.$$ (11)

We can use this weighting to form a new weighted objective function

$$\Omega =\{\delta \phi^{T}w^{T}w\delta \phi \}.$$ (12)

From Eq. (11), we minimize this objective function by

$$[{J_{w}}^{T}J_{w}+\lambda I]\Delta \mu_{i}={J_{w}}^{T}w\delta \phi_{i-1},$$ (13)

where $J_{w}=\mathrm{wJ}$.

Equation (10) finds the weighting matrix that minimizes the error between wJ and $J_{s}$. Larger values of $\lambda_{s}$ increase the error between the weighted and target sensitivities, which we discuss further in Sec. 4.2.1. As well as this, the optode arrangement itself limits how close to zero the residual between wJ and $J_{s}$ can get. With this methodology, we derive a weighting matrix from the sensitivity profiles of the optode arrangement that would allow us to be sensitive to a specific region in the sample defined as $J_{s}$. Other data types such as FD amplitude and phase and TD moments all amount to integral transforms of the original DTOF, and previous studies have investigated different ways to combine the sensitivity profiles to optimize diffuse optical measurements.^49^^–^^52^ Applying our derived weighting matrix to our measurements amounts to the same process. Rather than relying on thorough analytical analysis of the sensitivities or mathematical intuition to pick a favorable combination of data types, we present a numerical method that finds the combination of measurements wϕ with a sensitivity that most closely matches the desired sensitivity $J_{s}$.

This desired sensitivity may be some orthogonal imaging basis for tomography or a specific region of interest for continuous monitoring with diffuse optical measurements. With a careful choice of $J_{s}$, the step in Eq. (10) is an optimization step where we find the weightings of the measurements that maximize the sensitivity to a specific region for a given optode arrangement. For simultaneous retrieval of absorption and scattering coefficients or chromophore concentration, the same method can be applied using the corresponding sensitivity matrices. It is important to highlight that this weighting scheme relies only on calculating the sensitivity matrix of the optode arrangement.

## 3 Methods

All simulations were performed on a Windows 11 workstation with an Intel Core i7-14700K @ 3.40 GHz, with 64 GB of RAM and an NVIDIA RTX 4080 with 16 GB of VRAM using MATLAB 2025a.

### 3.1 Finite Element Solver

Our implementation of an FEM solver is written in MATLAB with supporting CUDA functions to solve Eq. (1) completely in parallel across mesh nodes and frequencies. ϕ is solved for using the biconjugate gradient-stabilized method with a factorized sparse approximate inverse pre-conditioner.^53^ This method of solving for the fluence has been shown to be extremely efficient on GPU hardware.^54^ In our implementation, we take advantage fact that

A(ωk)=A(ωo)+k·Im[A(ω1)], (14)

and that the sparsity of A and its pre-conditioner does not change among frequencies to reduce the memory bandwidth required to solve for all k frequencies in parallel. Using the truncated Fourier series approximation, this allows for fast computation of TD-gated data types.

The fluence was solved for a Dirac delta source over a 7-ns window with a 50-ps time resolution. This result was convolved with a Gaussian function with a 250-ps full width at half maximum (FWHM) to simulate a representative instrument response function (IRF). Our gating functions G(t) were defined as Gaussian gates with a 200-ps FWHM with a spacing of 200 ps. It has previously been shown that Gaussian gates give the best trade-off between temporal selectivity and computational complexity^38^; this allowed us to threshold k such that $\omega_{k}/2\pi <4.15\;\mathrm{GHz}$ (k≤30) and still get accurate results when compared with Monte Carlo simulations.

### 3.2 Meshes and Optode Arrangement

Mesh generation and manipulation were enabled by the iso2mesh package.^55^ This allowed for easy verification of our simulations with the mesh-based Monte Carlo package MMC.^9^

**Fig. 1:**
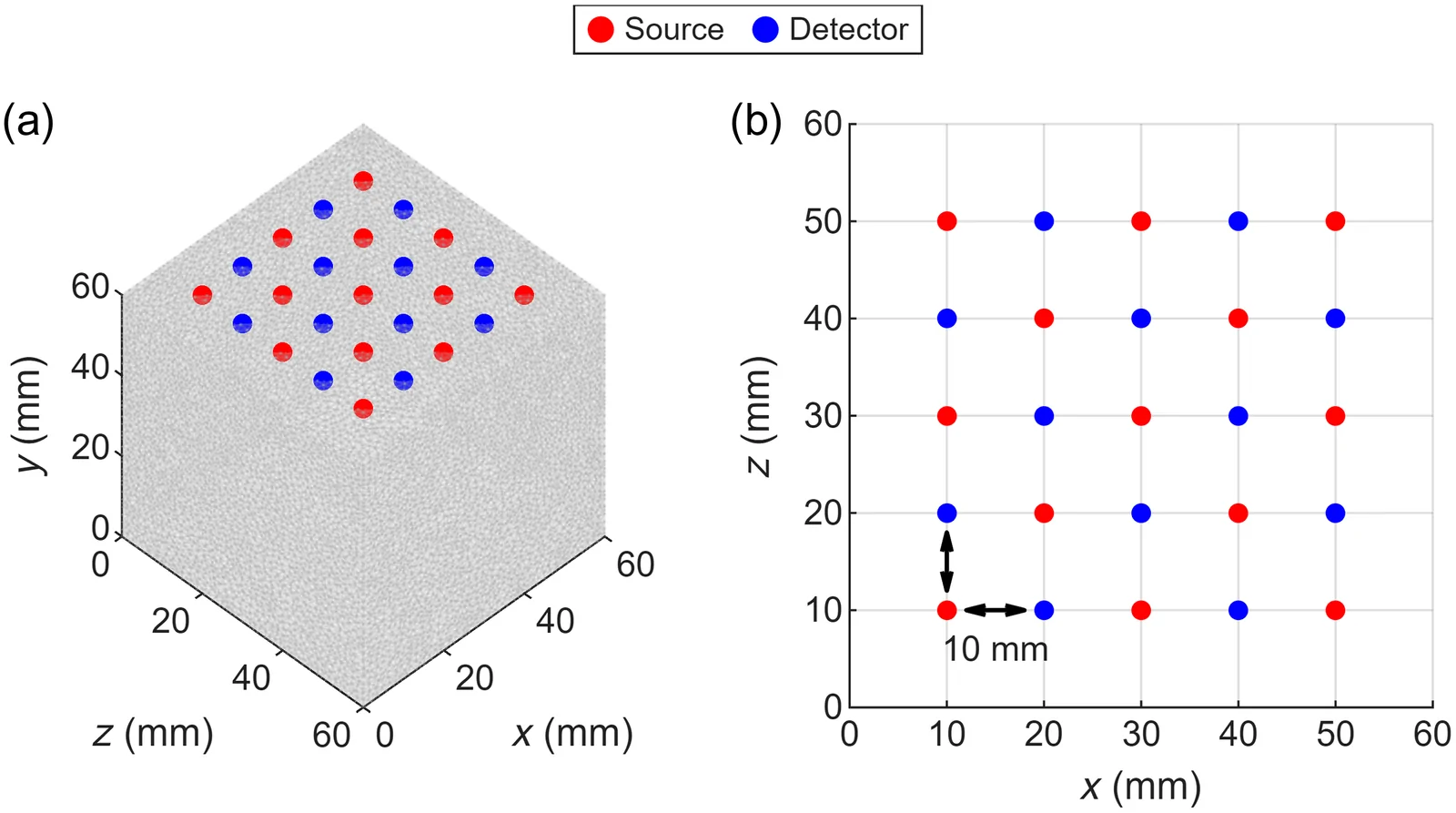
Domain and optodes used to generate forward data (a) and optode grid arrangement on surface (b). Source positions are shown in red and detector positions in blue.

The first simulations in this study were generated on a 60  mm×60  mm×60  mm cubic computational phantom. This domain was discretized into two tetrahedral meshes: a high-resolution “forward mesh” with 85,503 nodes and 511,623 elements and a lower-resolution “reconstruction mesh” with 68,797 nodes and 408,924 elements. Ground truth data for the reconstructions were generated on the forward mesh, and the LM minimization was performed using solutions from the reconstruction mesh. This was done to avoid biasing our DOT reconstructions using the same mesh (a.k.a inverse crime), as well as reducing compute times in the reconstruction. The optodes were placed in a bifurcated square grid of 13 sources and 12 detectors spaced by 10 mm. Each source was paired with every detector such that the minimum and maximum SDS were 10 and 50 mm, respectively, resulting in 156 channels each with 77 time gates to capture the whole DTOF. Figure 1 shows the meshed phantom and optode arrangement.

We only considered the changes in absorption in these studies, so the background optical properties were set to $\mu_{a}=0.008\;{\mathrm{mm}}^{-1}$, $\mu_{s}^{\prime }=0.9\;{\mathrm{mm}}^{-1}$, and n=1.4; these values were picked as they lie in the typical range of optical properties for breast tissue at 785 nm as reported in previous studies.^6^^,^^56^ The inclusion absorption was then set to $\mu_{a}=0.015\;{\mathrm{mm}}^{-1}$.

**Fig. 2:**
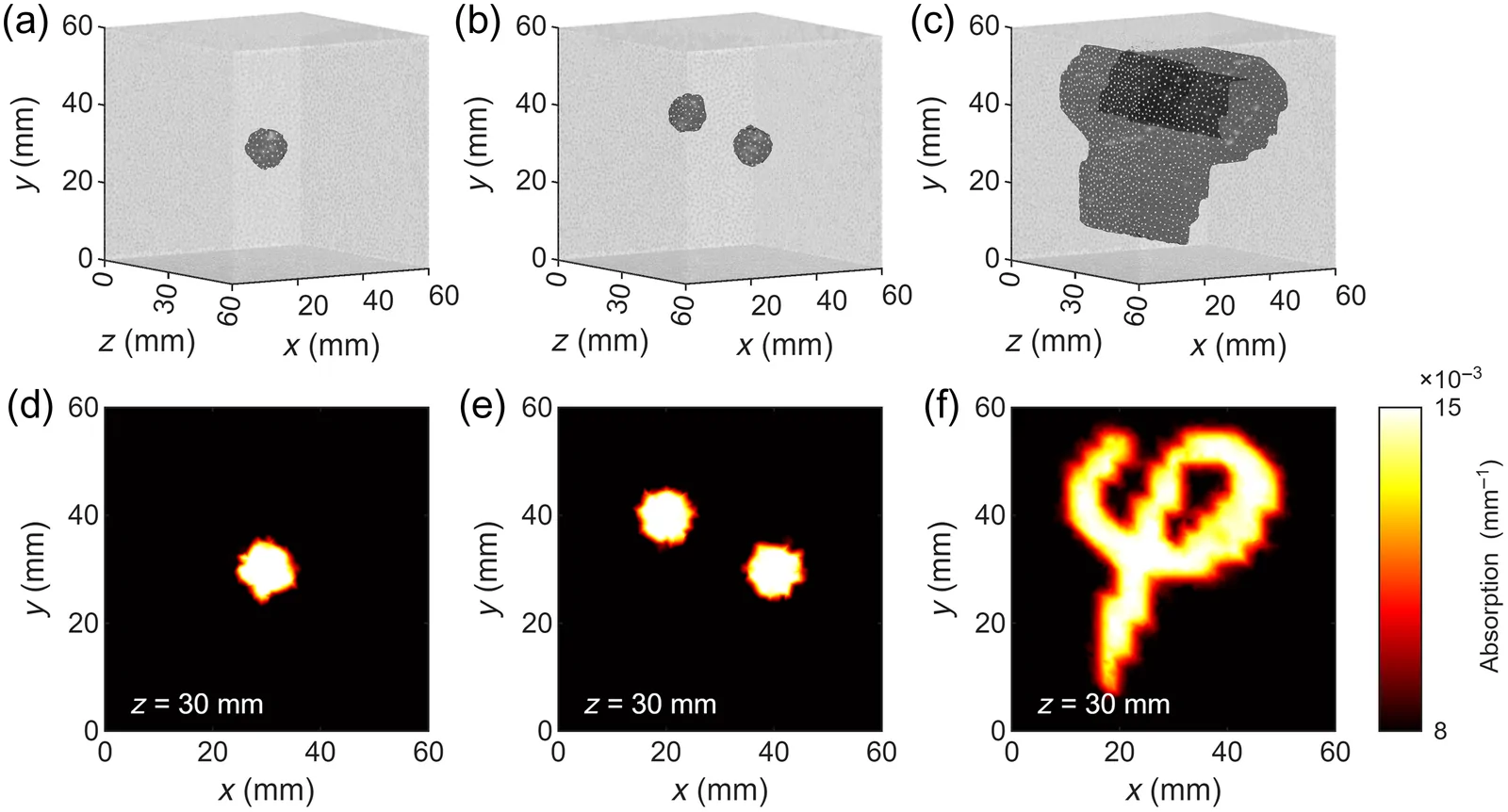
(a)–(c) Dice threshold isosurface of different ground truth absorptions used in reconstructions. (d)–(f) Two-dimensional (2D) slices of each distribution at z=30  mm with absorption in ${\mathrm{mm}}^{-1}$.

Three different sets of ground truths were used as shown in Fig. 2. A 10-mm-diameter spherical inclusion placed at different depths from 5 to 55 mm; two 10-mm spherical inclusions located at 20, 40, and 30 mm and 40, 30, and 30 mm; and finally, a phi-shaped inclusion. The single-inclusion reconstructions allow for the depth sensitivity of the reconstructions to be assessed. The other asymmetrical inclusions represent challenging imaging scenarios for reflectance DOT where the inclusions span a large depth range; this allows us to assess how robust our methodology is to significant changes in the ground truth. In addition, we generated ground truth data for the depth-varying inclusions using Monte Carlo fluence-generated with MMC to verify the model is robust to mismatch with the ground truth fluence. Monte Carlo simulations were generated with $10^{10}$ photons to minimize counting noise in the most attenuated channels.

**Fig. 3:**
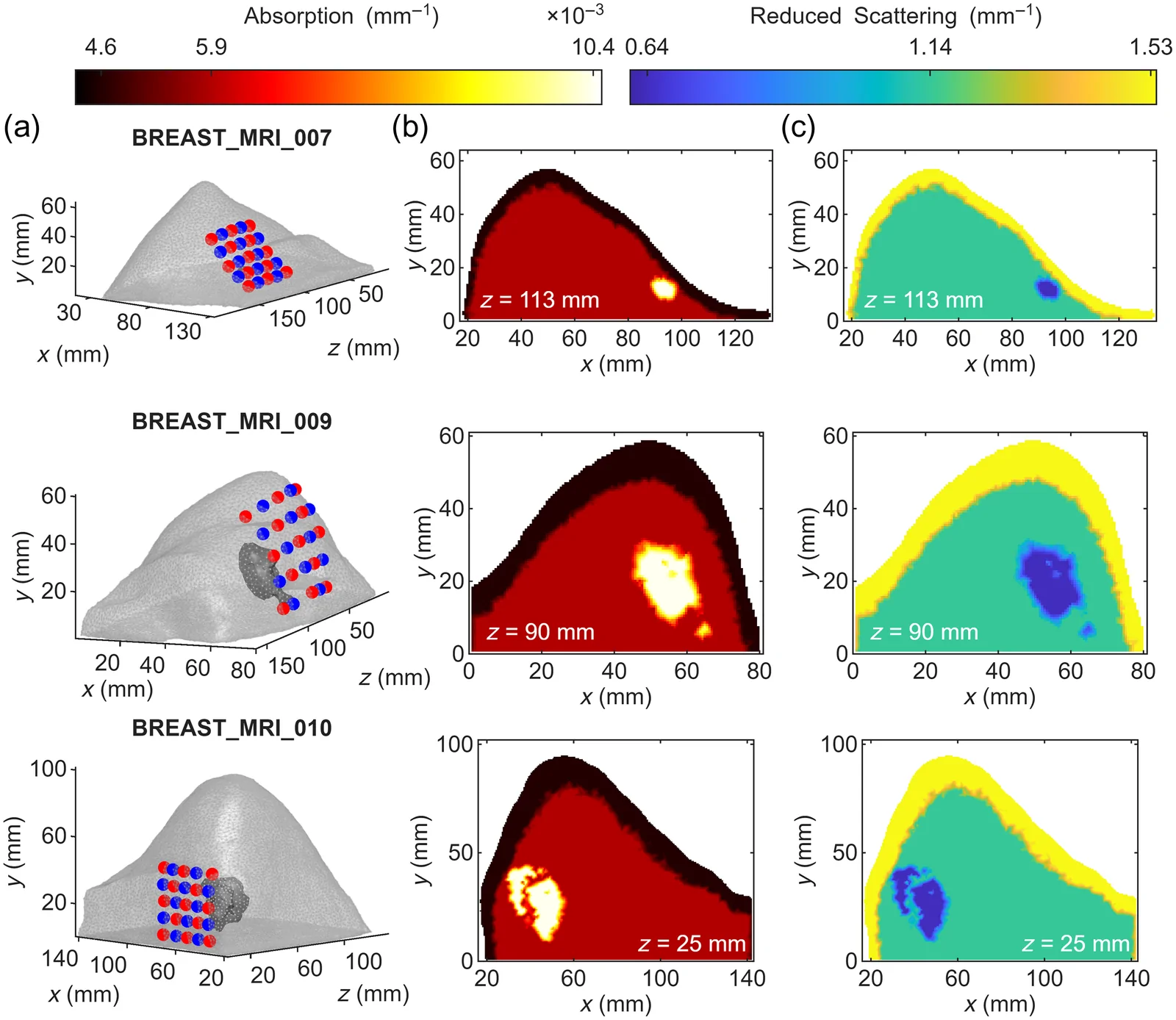
Various ground truths derived from the MRI dataset.^57^ (a) Meshes, inclusion locations, and optode arrangement in 3D. (b) 2D slice of the absorption at z=30  mm. (c) Reduced scattering on the same plane.

To further verify how robust our methodology is, reconstructions were performed on meshes generated from clinical MRI data taken at Duke University.^57^ The three datasets BREAST_MRI_007, BREAST_MRI_009, and BREAST_MRI_010 were used to generate the meshes shown in Fig. 3. Using a layered model of adipose, glandular, and cancerous tissue to approximate the ground truth, the MRI was segmented into these three regions. The absorptions of these regions were 0.0046, 0.0059, and $0.0104\;{\mathrm{mm}}^{-1}$, respectively. Likewise, the reduced scattering was set to 1.53, 1.1381, and $0.6369\;{\mathrm{mm}}^{-1}$. The same optode arrangement was projected onto the surface of these meshes near the inclusion location. Like with the cubic meshes, we defined separate forward and reconstruction meshes.

### 3.3 Measurement Noise

**Fig. 4:**
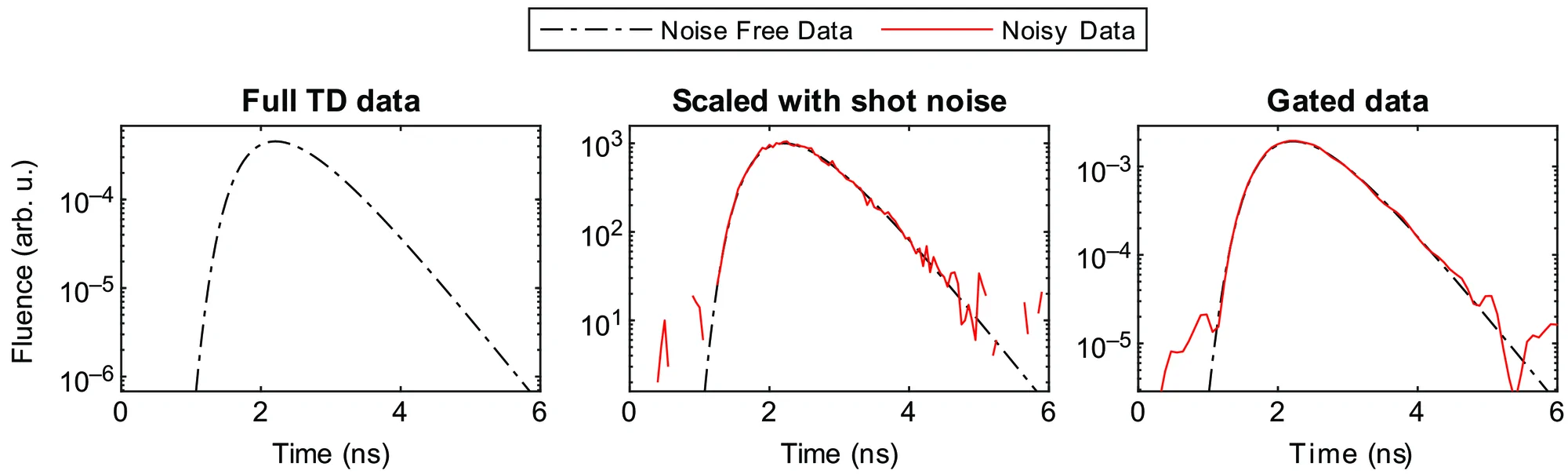
Noise simulation shown for the most attenuated channel. Noise-free data are shown as black dashed lines, and noisy data is shown as red solid lines.

TD-DOT techniques typically rely on time-correlated single-photon counting (TCSPC) to measure the DTOFs and, due to the high scattering of tissue, operate in photon-starved regimes. As such, the dominant sources of noise in these measurements are shot noise and detector dark counts. To simulate this noise, we generated the full time-domain data using the truncated Fourier series. These data are scaled to a set peak count and offset by a background dark count. After discretization, Poisson noise was generated using the new scaled DTOF and the dark count offset subtracted. Then, these data were scaled back down and gated with our gating functions G(t). Figure 4 shows the steps of this process.

Given that shot noise is dependent on the number of counts, we can quantify the performance of our algorithm relative to the peak counts in the most attenuated channel. For the noisy reconstructions, we scaled our simulation such that the maximum counts in the most attenuated channel logarithmically varied from $10^{2}$ to $10^{6}$, and the dark counts were set to 1%, 5%, and 10% of the peak counts in the same channel.

### 3.4 Gated Data Types and Sensitivities

In our LM minimization scheme, we look at the difference in the natural logarithms of the measurements such that our residual vector is defined as

$$\delta \phi =[\begin{array}{c} \ln(\phi_{1}^{M}(G_{1})-\ln(\phi_{1}^{C}(G_{1})\\ \ln\phi_{2}^{M}(G_{1})-\ln\phi_{2}^{C}(G_{1})\\ \vdots \\ \ln\phi_{NC}^{M}(G_{1})-\ln\phi_{NC}^{C}(G_{1})\\ \ln\phi_{1}^{M}(G_{2})-\ln\phi_{1}^{C}(G_{2})\\ \vdots \\ \ln\phi_{NC}^{M}(G_{NG})-\ln\phi_{NC}^{C}(G_{NG}) \end{array}],$$ (15)

where NC is the number of channels, NG is the number of time gates, and NM=NG·NC. For the noise-free data, the residual vector was filtered such that, for a given channel, we only used data that were above 1% of the maximum. This was picked so that reconstruction quality was not overestimated using data that could never be realistically measured in an experiment. For the noisy data, we introduced a secondary threshold at the square root of the dark counts above the dark count level. This threshold was picked, so all data used in the reconstruction were one standard deviation from the dark count level, reducing reconstruction bias to dark count noise. Finally, only data on the trailing edge at or below 80% of the maximum fluence were used in each channel for the reconstructions. The rationale of this choice is further discussed in Sec. 4.2.2.

**Fig. 5:**
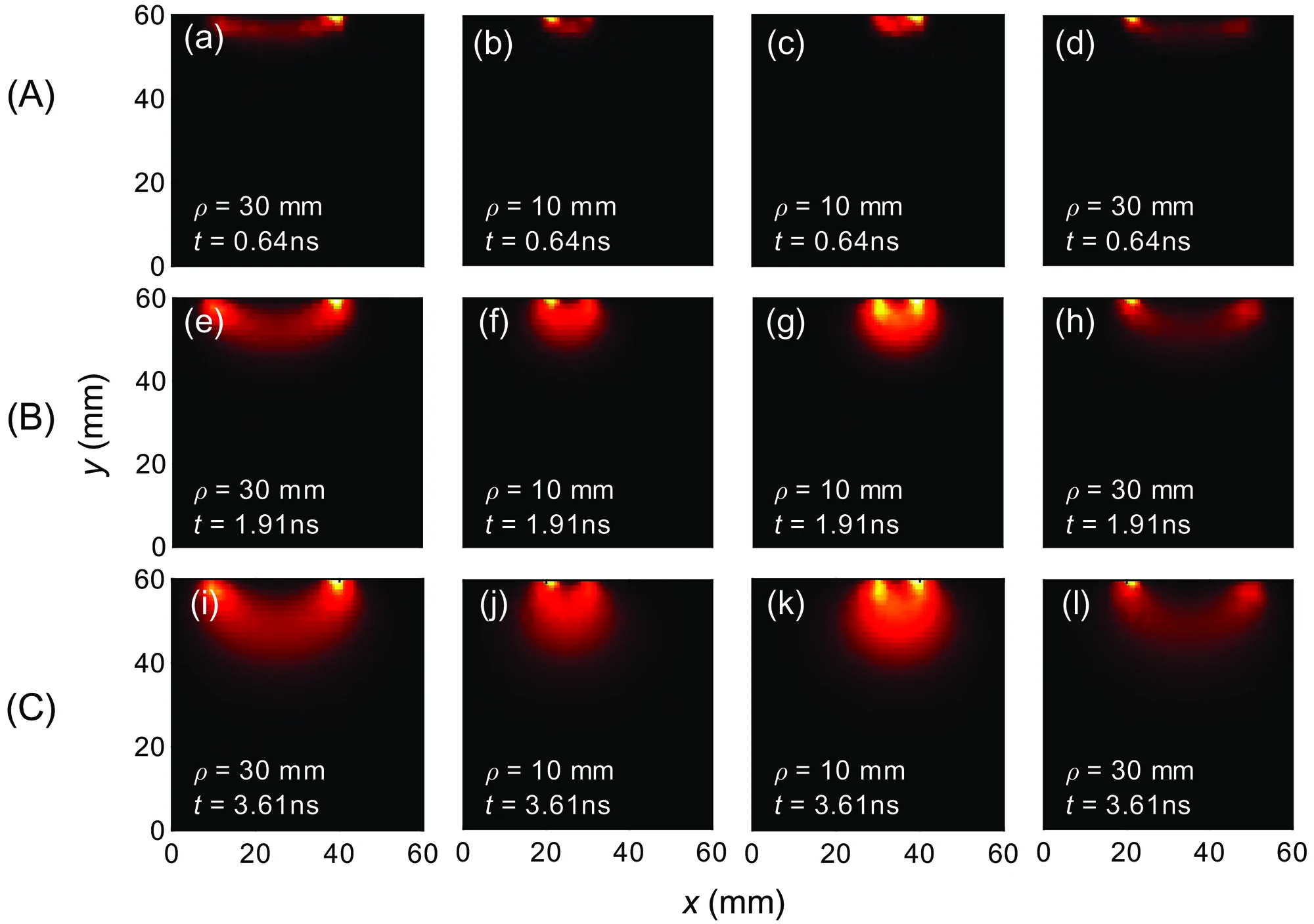
2D absorption sensitivity plots for different source detector separations (ρ) and time gates, calculated using the adjoint method. Sensitivities are shown for different gates: 0.64 ns (a), 1.19 ns (b), and 3.61 ns (c). All plots are normalized.

To generate the sensitivity matrix, the modeled fluence was interpolated to a voxel grid and formed using the adjoint method in the frequency domain,^58^ which was then transformed to the time-gated sensitivity by Eq. (5). The cubic meshes were interpolated to a 3  mm×3  mm×3  mm voxel basis (for a total of 9261 voxels), and the breast meshes were interpolated to a 4  mm×4  mm×4  mm voxel basis.

From Eq. (15), the corresponding sensitivity matrix is structured as

$$J=[\begin{array}{cccc} \frac{\partial \;\ln\;\phi^{C}}{\partial \mu_{1}} & \frac{\partial \;\ln\;\phi^{C}}{\partial \mu_{2}} & \ldots & \frac{\partial \;\ln\;\phi^{C}}{\partial \mu_{NV}} \end{array}],$$ (16)

where NV is the number of voxels in the grid, and $\frac{\partial \;\ln\;\phi^{C}}{\partial \mu_{i}}\in R^{\left(NM\times 1\right)}$. Figure 5 shows some select sensitivities at z=30  mm for different time gates.

### 3.5 Weighted Reconstruction Algorithm

The optical properties were retrieved by minimizing Eq. (12) using the iterative scheme defined in Eq. (13). The residual was formed from the forward mesh and reconstruction mesh fluences. The sensitivity matrix, weightings, and optical property updates were calculated on the voxel grid basis, and the residual was updated by interpolating the optical property update back onto the reconstruction mesh basis.

For the cubic phantom reconstructions, the initial optical property guess on the reconstruction mesh was set to the forward mesh background; for the breast reconstructions, the initial property guess was set to the adipose tissue properties. λ was set to $10^{2}$ for all reconstructions. Equation (13) was iterated until the error was less than 0.5% or the projection error changed by less than 2%.

For the cubic mesh, our weightings were found by choosing the target sensitivity matrix $J_{s}$ to be an array of 5832 3D Gaussian spots with a 1.5-mm FWHM. Such that $J_{s}\in R^{5,832\times NV}$ where each row is a Gaussian centered at every voxel within a 1.5-mm perimeter of the mesh boundary. An ideal choice of $J_{s}$ would form an orthonormal basis such as a Dirac delta at each voxel. As the light is so highly scattered, we see that the sensitivities for each channel have a very limited span of spatial frequencies which in turn limit the achievable resolution of wJ. Our choice of $J_{s}$ was found to be a good compromise, where each spot is centered on one voxel and the FWHM was set to half the voxel grid resolution to minimize overlap among the matrix rows.

**Fig. 6:**
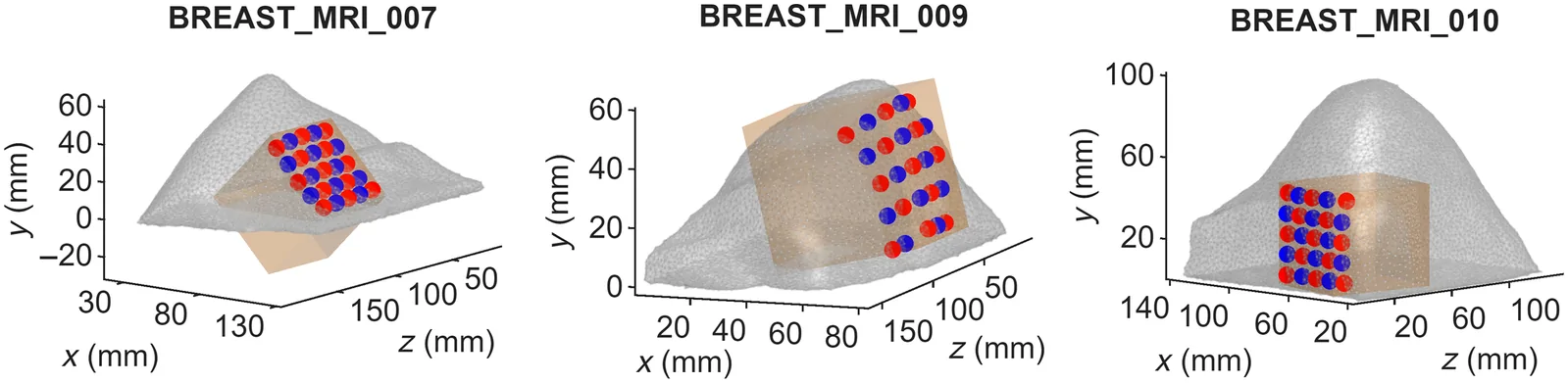
Breast reconstruction mesh and their 3D ROIs.

Following this logic, $J_{s}$ was defined similarly for the breast meshes. A cuboid region of interest (ROI) was defined with the top plane orthogonal to the central optode normal and a width of ∼60  mm (depending on the optode projection onto the surface). The ROI spanned 50 mm into the mesh along the optode normal. $J_{s}$ was chosen such that the Gaussian spots were centered on each voxel in this ROI with a 2-mm FWHM, half the voxel grid resolution. Any voxels outside of the mesh or closer than 0.5 mm to the mesh boundary were excluded. Figure 6 shows the ROI for each breast mesh. As we are reconstructing both absorption and scattering, our two property sensitivity matrices are structured as

$$J_{s[\mu_{a},\mu_{s}^{\prime }]}=[\begin{array}{cc} J_{s} & 0\\ 0 & J_{s} \end{array}].$$ (17)

This choice of $J_{s}$ minimizes the crosstalk among optical properties while still enhancing their individual spatial selectivities.

**Fig. 7:**
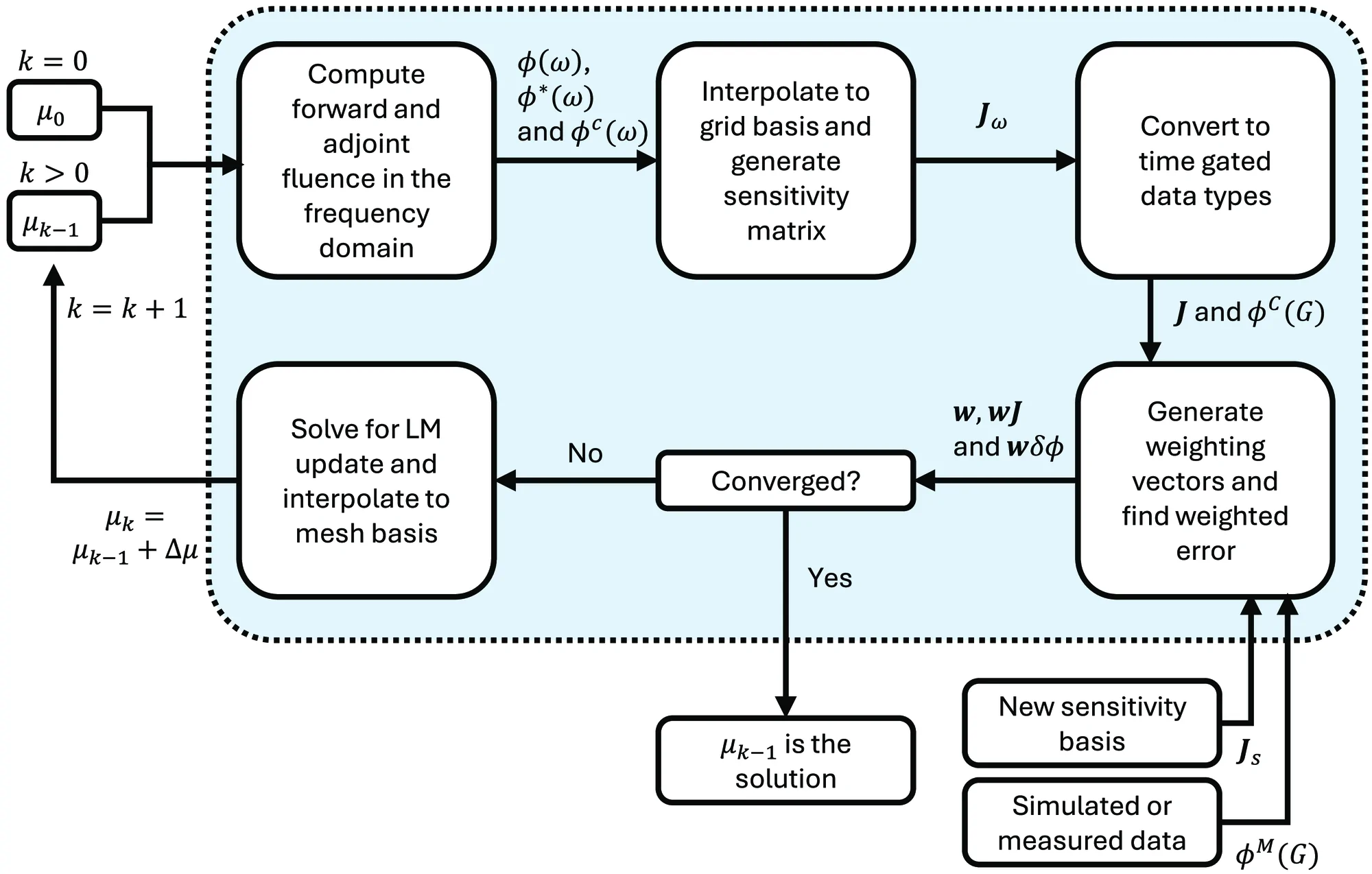
Flow chart of the reconstruction process.

Figure 7 shows a flowchart of our weighted reconstruction process. At each iteration, the weighting matrix w was recalculated as the update to the optical proprieties correspondingly changes J.

For the noise-free simulated data, $\lambda_{s}=10^{-6}$. For different noise levels, the regularizer to find the weightings $\lambda_{s}$ was tuned between $5\times 10^{-5}$ and $5\times 10^{-2}$ as shown in Table 1; these values were found empirically. Due to the large source detector separation in some channels, there is a significant Poisson noise in the Monte Carlo data; we found that setting $\lambda_{s}$ to $10^{-4}$ resulted in good reconstructions with good depth sensitivity. The regularizers λ and $\lambda_{s}$ are scaled by the maximum value of the Hessian matrix diagonal such that $\lambda \to \lambda \cdot \max[\mathrm{diag}\{{J_{w}}^{T}J_{w}\}]$ and $\lambda_{s}\to \lambda_{s}\cdot \max[\mathrm{diag}\{JJ^{T}\}]$.

**Table 1:** Our choice of $\lambda_{s}$ values for various peak counts.

Peak counts	$10^{2}$	$10^{3}$	$10^{4}$	$10^{5}$	$10^{6}$
Regularization, $\lambda_{s}$	$5\times 10^{-2}$	$5\times 10^{-3}$	$5\times 10^{-4}$	$1\times 10^{-4}$	$5\times 10^{-5}$

A version of the MATLAB package used to generate the FEM solutions and reconstructions is available at https://github.com/tanner-lab/DOGPUP.

### 3.6 Reconstruction Metrics

To assess the reconstructions in the cubic mesh, we used three metrics; the root mean square error (RMSE) defined as

$$\mathrm{RMSE}=\sqrt{\frac{1}{n}\overset{n}{\underset{i=1}{\sum }}\left[\mu_{i}-{\overset{¯}{\mu }}_{i}\right]},$$ (18)

contrast ratio (CR) to quantify the contrast in the image,

$$\mathrm{CR}=\frac{\text{mean}[{\overset{¯}{\mu }}_{\mathrm{inc}}]\cdot \text{mean}[\mu_{bg}]}{\text{mean}[{\overset{¯}{\mu }}_{bg}]\cdot \text{mean}[\mu_{\mathrm{inc}}]},$$ (19)

and the Dice–Sørensen coefficient to quantify image similarity,

$$\text{Dice}=\frac{2\left|S(\mu )\cdot S(\overset{¯}{\mu })\right|}{\|S(\mu ){\|}_{1}^{2}+\|S(\overset{¯}{\mu }){\|}_{1}^{2}},$$ (20)

where $\overset{¯}{\mu }$ is the reconstructed optical property, and μ is the ground truth. S(μ) is a function that thresholds the absorption to a binary distribution such that any value over $\frac{1}{2}(\text{median}[\mu ]+\max[\mu ])$ is 1 and otherwise 0. The same threshold taken from the ground truth is used to delineate between the background and the inclusion in the CR expression. For the highly heterogeneous breast meshes, we used the Pearson correlation coefficient to quantify the image similarity, as the ground truth is no longer binary. This is defined as

$$\rho_{\mu ,\overset{¯}{\mu }}=\frac{\mathrm{cov}(\mu ,\overset{¯}{\mu })}{\sigma_{\mu }\sigma_{\overset{¯}{\mu }}},$$ (21)

where $\sigma_{\mu }$ and $\overset{¯}{\mu }$ are the standard deviations, and $\mathrm{cov}(\mu ,\overset{¯}{\mu })$ is the covariance. To compute our metrics for the layered breast reconstructions, we only selected the subset of nodes in the ROI.

## 4 Results

### 4.1 Model Verification

**Fig. 8:**
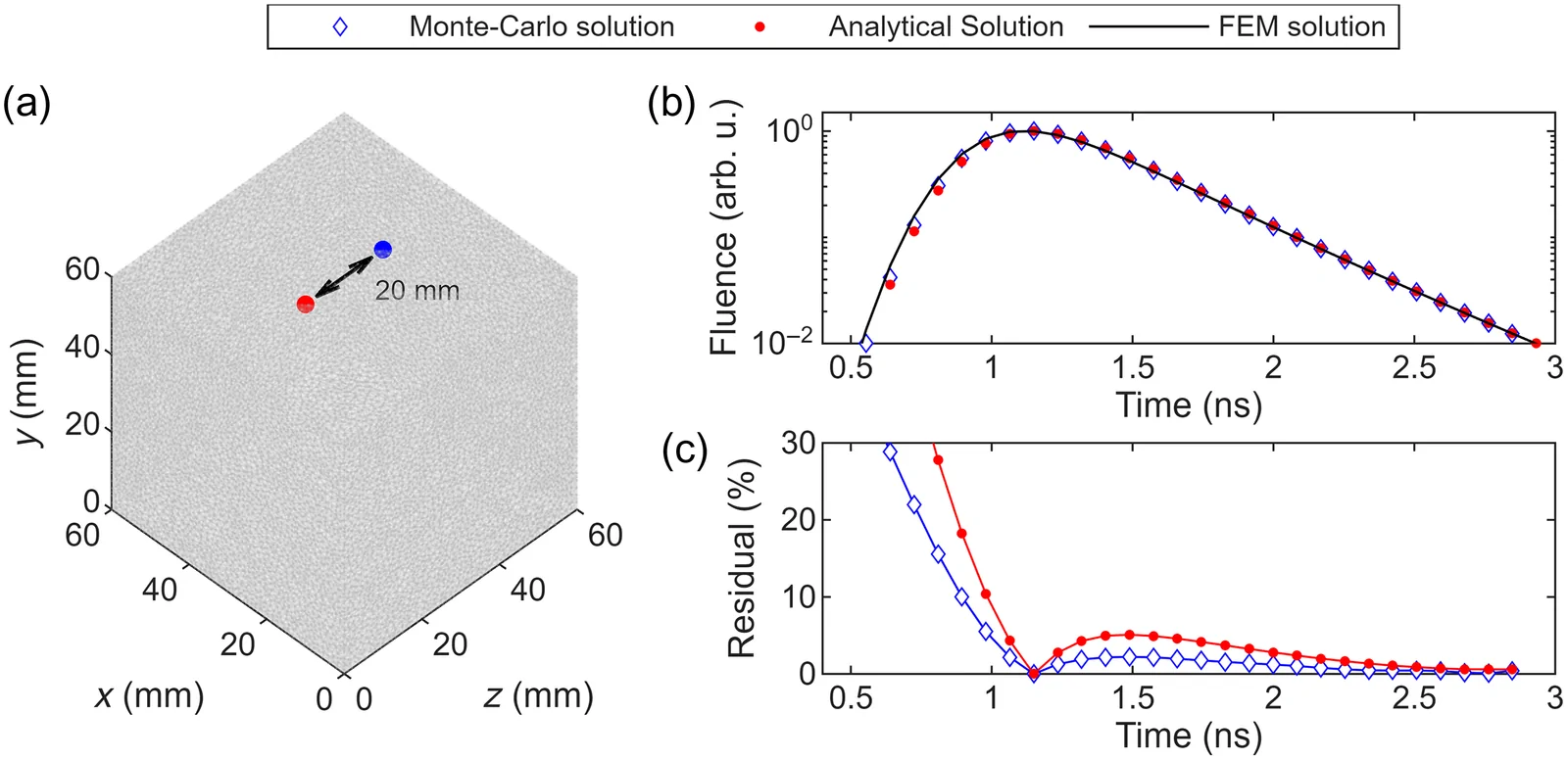
Computational phantom and source detector separation (a). Modeled fluence (b) and residual (c). Monte Carlo solution shown as open blue diamonds, analytical solution shown in red filled circles, and FEM solution as a black line.

To verify the accuracy of our model, we used the forward mesh with an SD pair placed centrally on the top facet of the mesh with an SDS of 20 mm. Figure 8 shows our model compared with the analytical solution of the diffusion equation for a semi-infinite slab^59^ and the Monte Carlo solution generated using MMC^9^ all gated with our gating functions G(t).

The larger deviation from the analytical model is expected as the simulated mesh is relatively small. The FEM solution has good correspondence with the MMC solution with an $R^{2}$ of 0.9981 and 0.9978 for the fluence and log fluence, respectively. The residual between the MMC and FEM solutions is poor for the small values on the leading edge, but at 85% of the signal, it drops below 5.5% and remains below 2.25% on the falling edge. We highlight that these early gate residuals are typical of comparisons between Monte Carlo and FEM solutions,^60^ and as the natural logarithm of the DTOFs is used in the reconstruction process, this discrepancy becomes less significant. In addition, as we show in Secs. 4.2 and 4.3, using only late gates in the analysis still gives high-quality results in reflectance geometry.

### 4.2 Sensitivity Weighting

#### 4.2.1 Regularizing the weightings

**Fig. 9:**
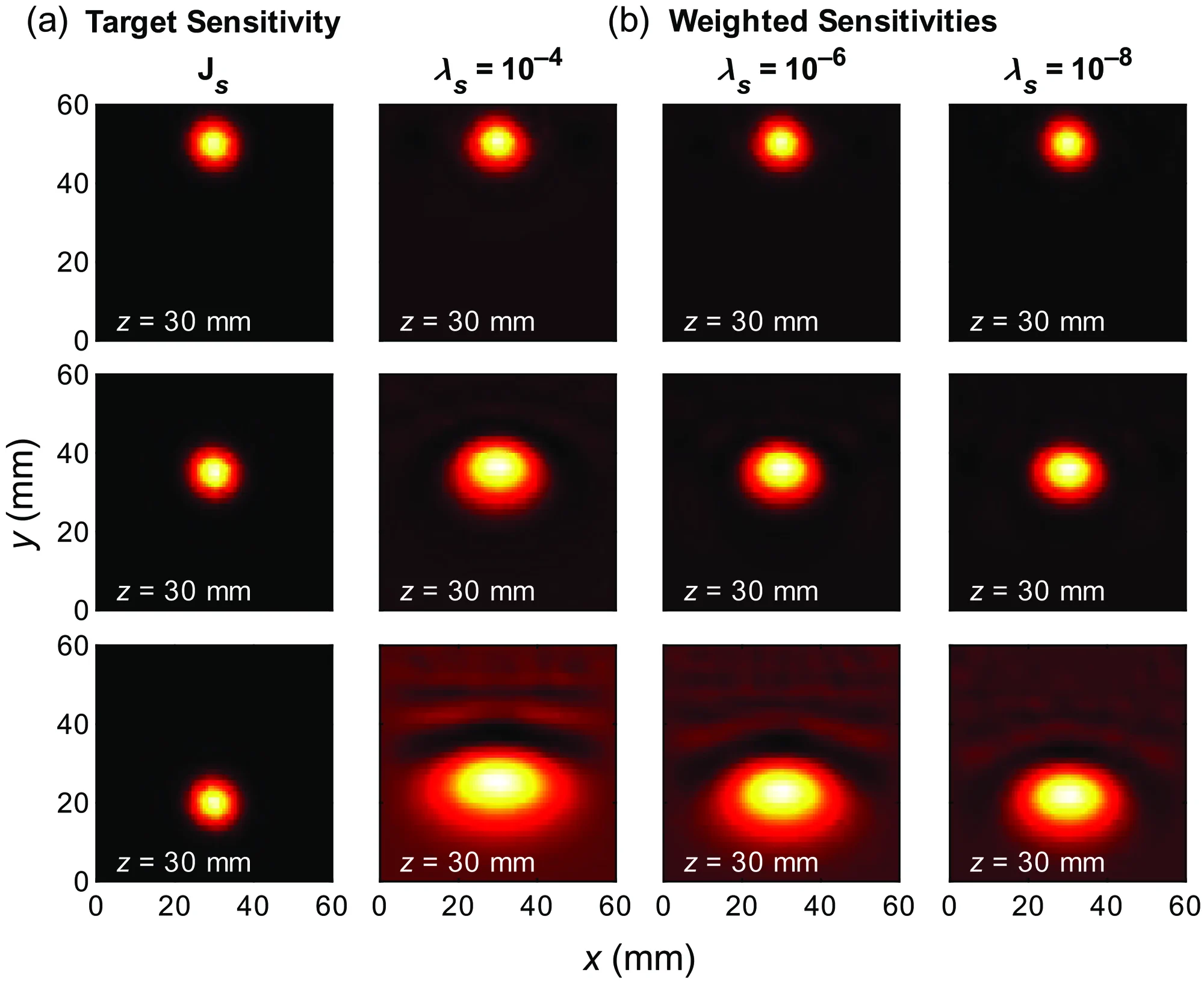
Target (a) and weighted (b) sensitivities at z=30-mm slice though mesh, at different locations (50, 35, and 20 mm) for different regularizations. All plots are normalized.

In Eq. (10), the regularizer $\lambda_{s}$ is used to stabilize the solution and smooth the weighting matrix w. Large $\lambda_{s}$ corresponds to smoother solutions but reduces the quality of the reformatted sensitivity $J_{w}$ compared with the target sensitivity $J_{s}$. From Fig. 9, we can see that depending on the magnitude of the regularizer, the weighted sensitivity is limited in the depth of the spot it can form. A small regularization reduces the error between $J_{w}$ and $J_{s}$ deeper into the sample. As none of the parameters in Eq. (10) depends on experimentally measured parameters, the inversion is stable for very small regularizations.

**Fig. 10:**
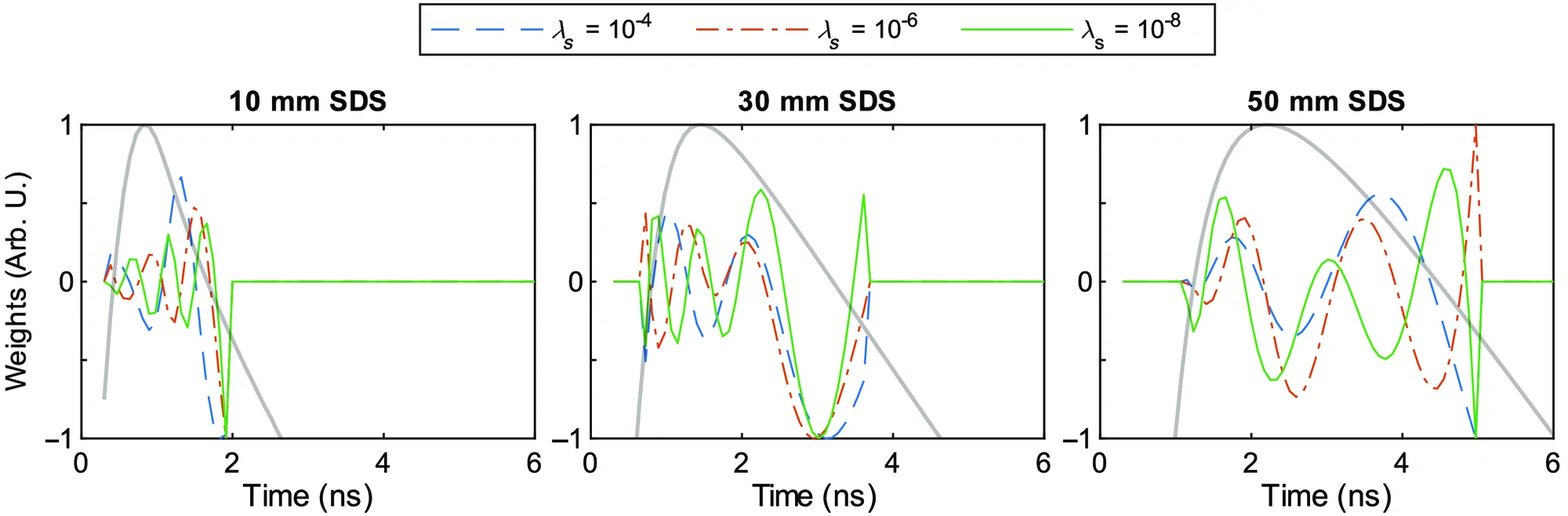
Weightings to form a spot located at 30, 35, and 30 mm from select channels. Fluence in each channel is shown in gray.

Previously reported normalization schemes involve applying some weighting parameter across the nodes in the sensitivity matrix to tend the reconstruction toward deeper nodes.^27^^,^^36^^,^^37^ Correct tuning of this parameter requires knowledge of the ground truth and can result in over-fitting at deep nodes. Like these normalization schemes, we now have an extra parameter to tune. Compared with the parameters based on node depth, tuning of $\lambda_{s}$ is much more intuitive and no prior information of the scene or depth calibration is needed. As we can see in Fig. 10, a smaller $\lambda_{s}$ value results in higher-frequency solutions, so these weighted measurements are more sensitive to noise. $\lambda_{s}$ must be chosen such that noise does not dominate the new weighted residuals, and it is left to the user to strike the balance such that depth sensitivity is maximized for the level of noise in the measurements.

The quality of $J_{w}$ compared with $J_{s}$ is dependent on the optode arrangement used. Due to the dense optode arrangement simulated in this study, the resulting large spatial overlap in sensitivities, and the depth selectivity in the gates, we can form spots of high fidelity. For sparse optode arrangements, this is not necessarily true. The choice of $J_{s}$ is not limited to just a spot, and for a given optode arrangement, the ideal $J_{s}$ and the following derived weightings will be different. We highlight that the main value of this technique is the ability to optimize the measurement basis for a given optode arrangement and region of interest.

#### 4.2.2 Enhancing spatial selectivity

**Fig. 11:**
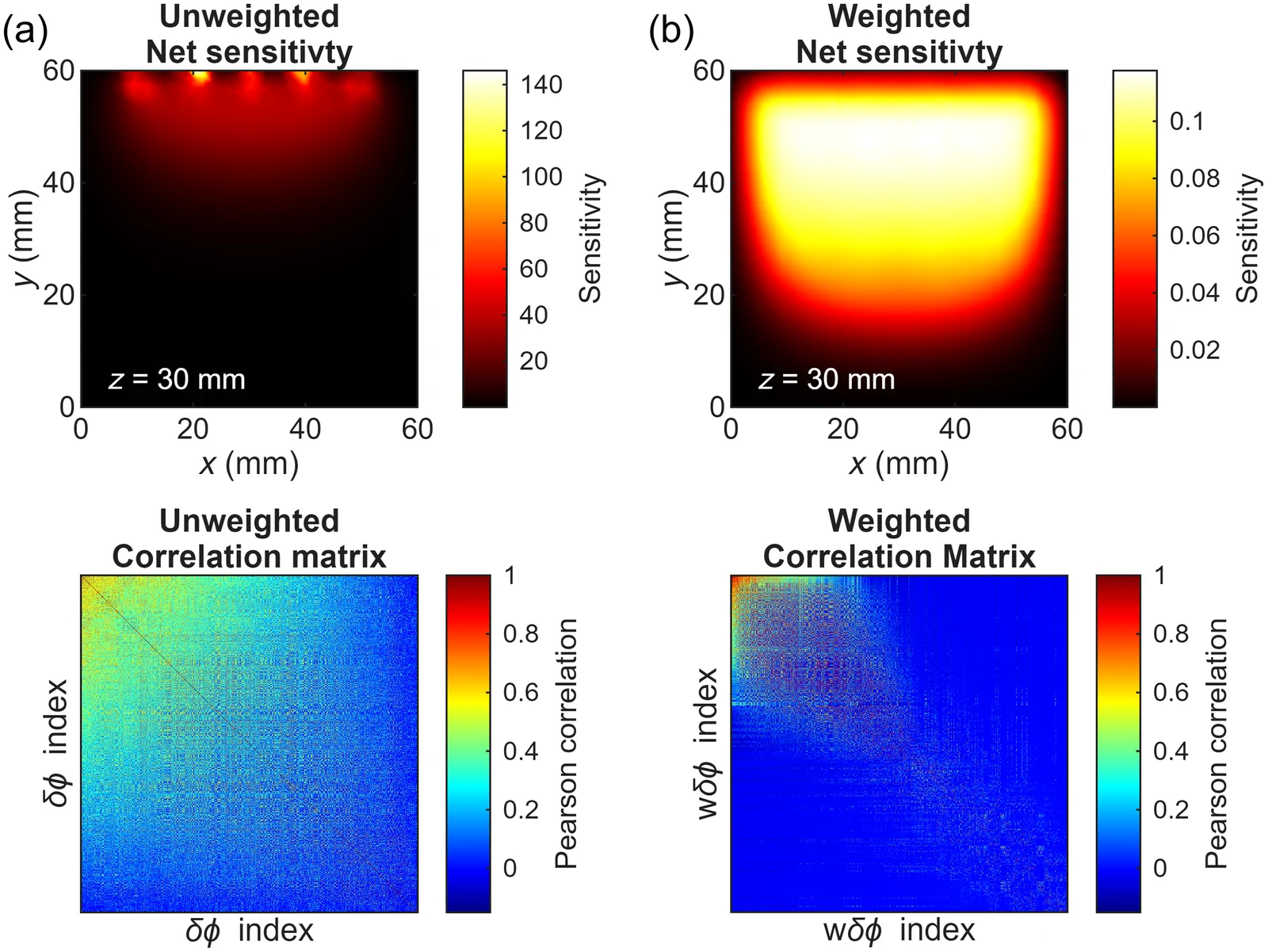
Net sensitivity and correlation of each row in the sensitivity matrix for the unweighted (a) and weighted (b) sensitivities.

To verify that our weighting scheme does form the expected sensitivities, we calculated the residual using the fluence from the forward mesh with a spherical absorbing inclusion at 30, 30, and 30 mm. Our residual was formed by the difference among the measurements on the forward mesh and homogenous reconstruction mesh. The regularizer to find the weightings for the sensitivity matrix was set to $\lambda_{s}=10^{-5}$. Figure 11 shows a comparison of the net sensitivities defined as

$$\|J\|=\sqrt{\overset{NM}{\underset{i=1}{\sum }}{\left[\frac{\partial \;\ln\;\phi_{i}^{C}}{\partial \mu }\right]}^{2}},$$ (22)

for both the unweighted and weighted sensitivity as well as the spatial correlation between each row of the sensitivity matrices. We can see the the weighting flattens the net sensitivity deep into the sample while reducing the spatial correlation between each matrix row. By doing this, the sensitivity at deep nodes is enhanced without reducing the sensitivity at the surface, unlike other depth normalization schemes. We also reduce the redundancy in our measurements as each weighted combination of measurements is only sensitive to a small region with less overlap. The limit of the high sensitivity region in the weighted net sensitivity is dependent on the maximum SDS, timing gates used, and the regularizes $\lambda_{s}$.

**Fig. 12:**
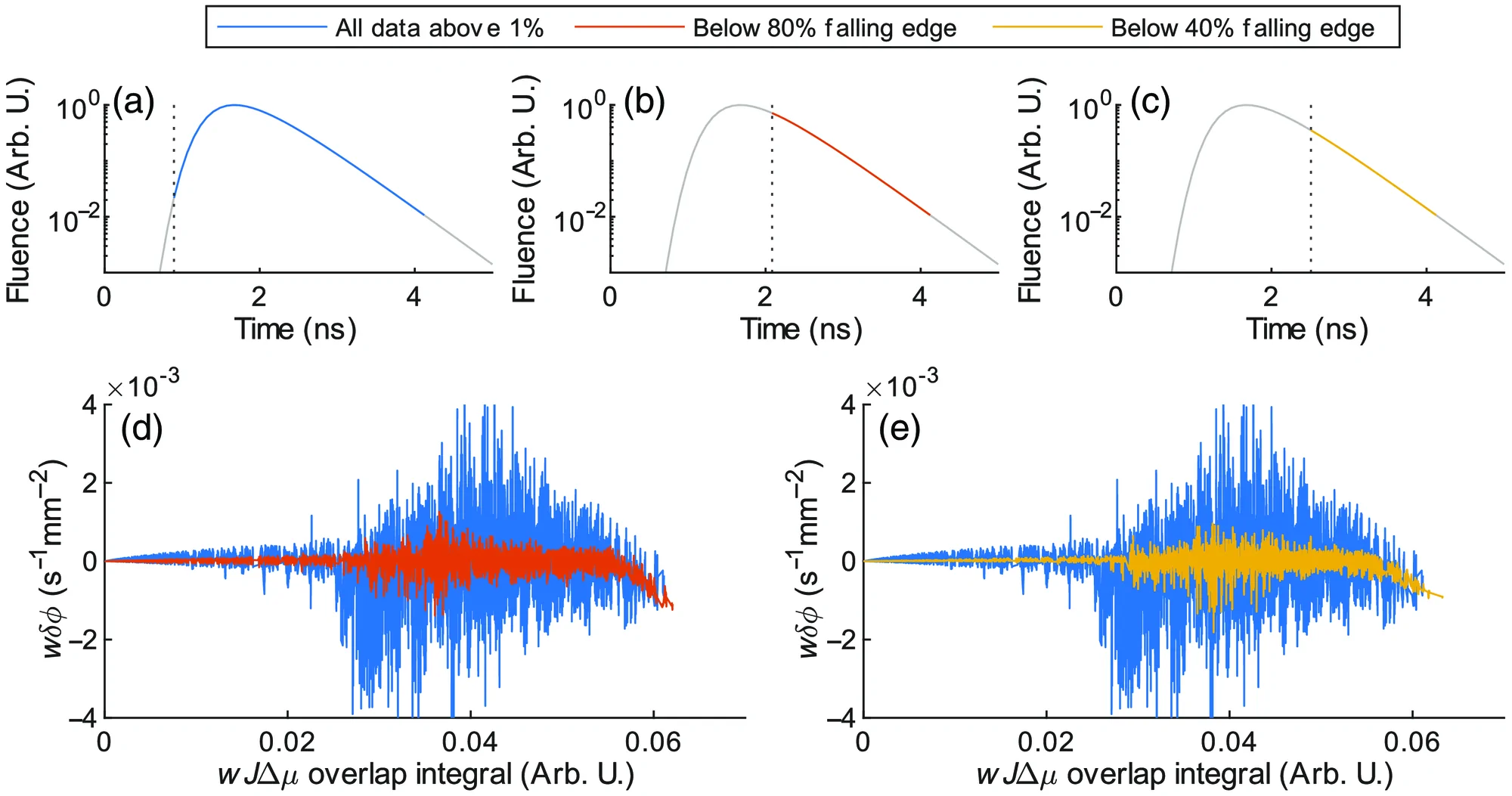
Visualizations of the data used to form the weighted residual vector: data above 1% (a, blue), data from 80% on the trailing edge (b, red) and data from 40% on the trailing edge (c, yellow), as well as the weighted residual calculated from all data above 1% (d and e, blue), data from 80% on the trailing edge (d, red) and 40% on the trailing edge (e, yellow).

If our weighted sensitivities have the localization and reduced correlation we think they do, the minimum element of the weighted residual should correspond to the sensitivity with the largest overlap integral with the inclusion. Figure 12 verifies this as we can observe a clear minimum in the weighted residuals when the overlap of the weighted sensitivity is maximized. The noise we see in the weighted residual calculated using all of the gated data (shown in blue) is computational noise from mismatched boundary geometry between the forward and reconstruction meshes. This results in a large discrepancy between the early gate measurements and resulting in a global minima that does not follow this trend. Hence, why using only data from below 80% and 40% the maximum on the falling edge reduces this noise, resulting in the expected global minima. As throwing away as little data as possible is preferable, for all our following reconstructions, the trailing edge threshold is set at 80%.

### 4.3 Reconstructions

#### 4.3.1 Depth and reconstruction quality

**Fig. 13:**
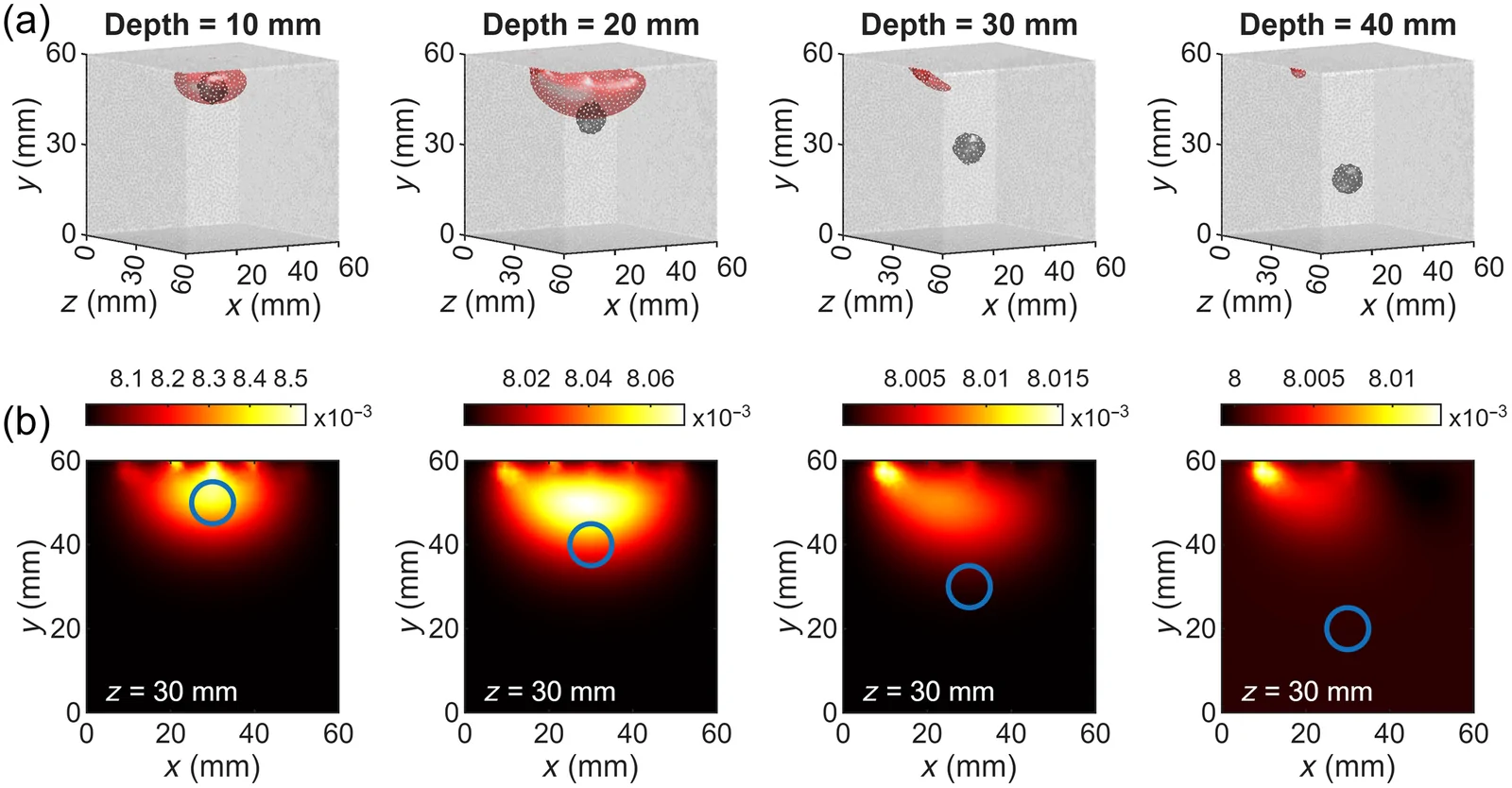
Unweighted reconstructed and ground truth absorptions. (a) Dice threshold isosurface of the ground truth absorption (gray) and reconstructions (red) for different depths. (b) 2D slices of the reconstructed absorption in ${\mathrm{mm}}^{-1}$ at z=30  mm. The true inclusion location is indicated by the blue circle.

To assess the depth sensitivity of our scheme, we reconstructed the absorption profile of the spherical inclusions at 11 depths from 5 to 55 mm, using both the unweighted and weighted scheme. Weighted reconstruction was also performed using Monte Carlo ground truth data to assess the effects of model mismatch on our method. As shown in Fig. 13, the unweighted scheme results in very poor inclusion localization beyond 10 mm. This is due to the extremely high sensitivity near the optodes biasing the reconstruction. As the inclusion goes deeper, the reconstruction begins to bias toward optodes at the periphery; these optodes have a larger average SDS, so measurements taken from here are sensitive to deeper regions in the sample. This indicates that our reconstruction is sensitive to these deep inclusions despite the bias toward the optodes. Typically, this can alleviated by excluding nodes near the surface in the reconstruction or depth-based normalization of the sensitivities, but these normalization schemes rely on some prior knowledge of the inclusion.

**Fig. 14:**
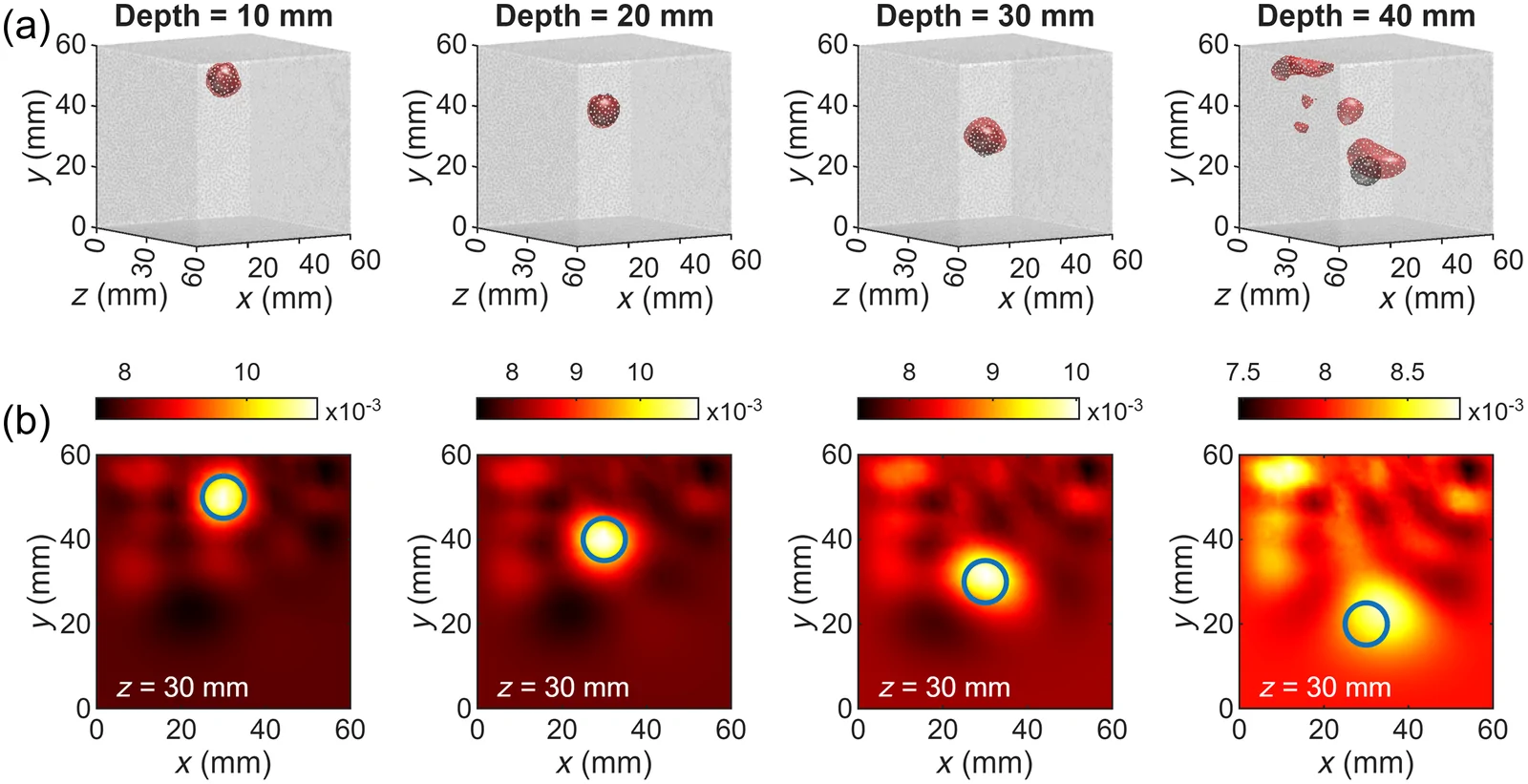
Weighted reconstructed and ground truth absorptions. (a) Dice threshold isosurface of the ground truth absorption (gray) and reconstructions (red) for different depths. (b) 2D slices of the reconstructed absorption in ${\mathrm{mm}}^{-1}$ at z=30  mm. The true inclusion location is indicated by the blue circle.

**Fig. 15:**
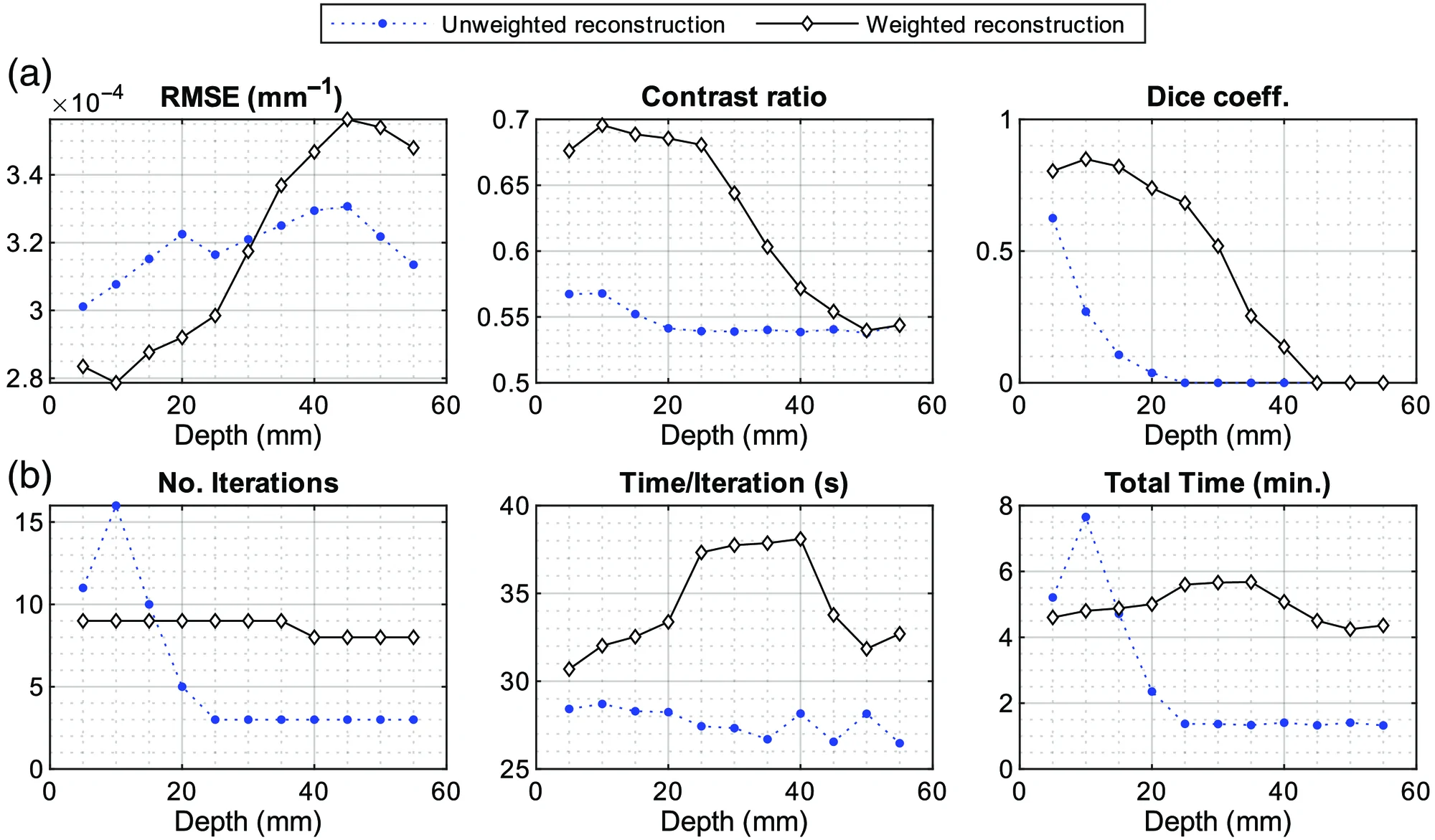
RMSE, contrast ratio, and Dice–Sørensen coefficient for absorption reconstructions plotted against inclusion depth (a). Reconstruction timing and iteration counts (b). Unweighted reconstructions shown in dashed blue line and weighted reconstructions shown in solid black with open diamonds.

Figure 14 shows the results using our weighted reconstruction scheme [Eq. (13)]. We can see the weighted reconstruction is able to accurately localize inclusions up to 40 mm, although with a reduction in contrast. This improvement in performance is due to the flattening of the net sensitivity across the depth of the sample when we weight our sensitivities to form the new basis wJ as shown in Fig. 11. From this plot of the net sensitivity, we see at 40 mm deep the sensitivity begins to taper off, which is why we see the noisy result at 40 mm. It is important to highlight that sensitivity to deep inclusions with reflectance measurements has been shown previously,^36^^–^^38^ but these reconstructions underestimate the depth without priors that rely on some inference of the inclusion depth unlike our scheme.

From Fig. 15, we see quantitatively that our weighted scheme dramatically improves reconstruction quality. Beyond 30-mm inclusion depth, the RMSE of our weighted reconstructions is worse, despite the improvement to the contrast and similarity. As we are not biased to the surface sensitivities, our reconstructions are more noisy, but on the whole, the RMSE is not high and the inclusion is easily resolvable up to 40 mm. Our implementation of the weighting scheme results in an extra 2 to 10 s per iteration compared with the unweighted scheme. For the shallow inclusions (<20  mm), where both unweighted and weighted algorithms resolve the inclusion, the weighted algorithm still converges more quickly. Across the whole depth span, we see more consistent performance in the weighted algorithm. We separately observed that the difference in memory usage at any one time was negligible between the two algorithms, with both having a peak usage of 5.59 GB. As the sensitivity matrix is already very large due to the number and channels and gates, the weighted sensitivity matrix and corresponding weighted measurements were of a similar size. All timing and iterations for every reconstruction can be found in the Supplementary Material.

**Fig. 16:**
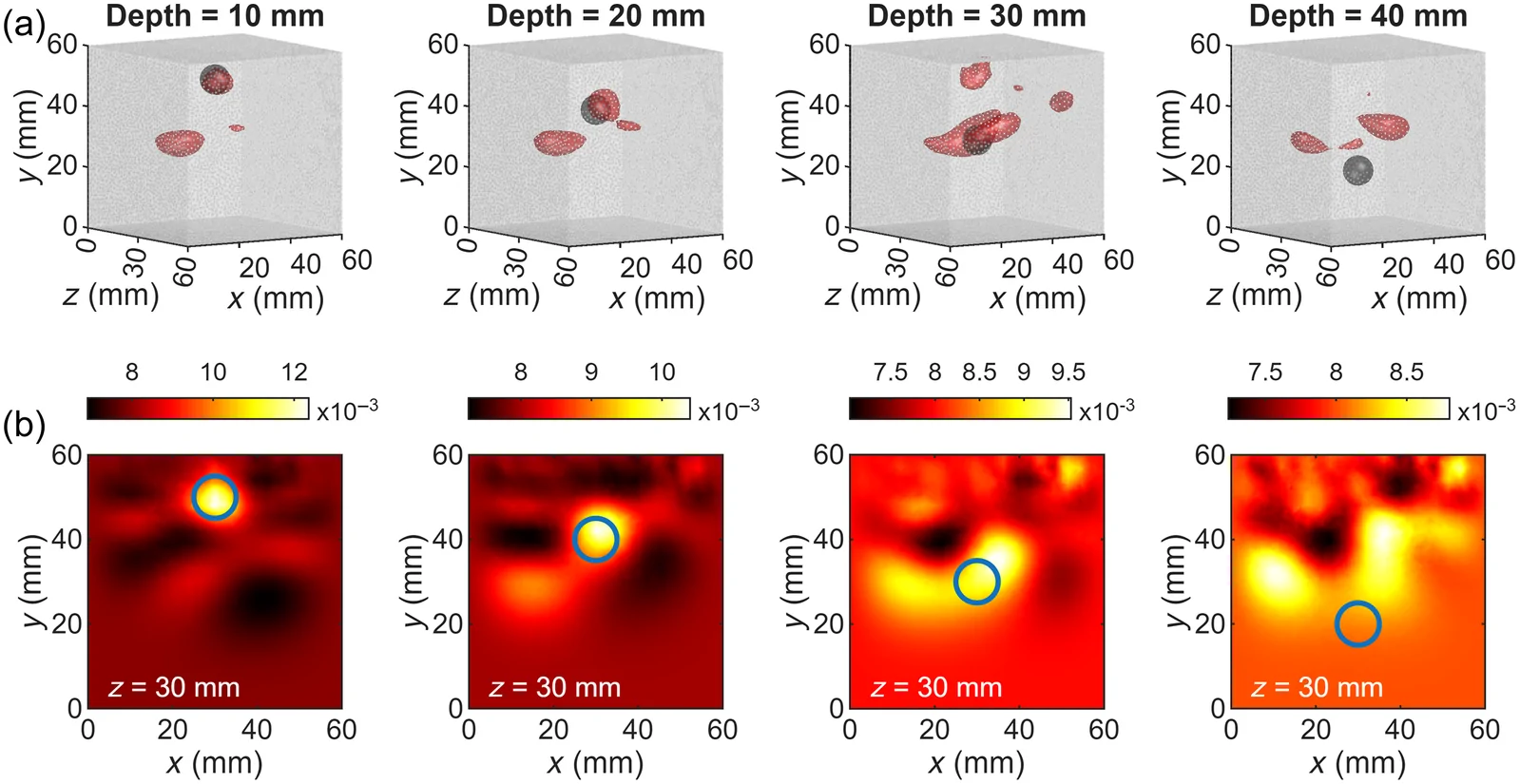
Weighted reconstructed and ground truth absorptions for Monte Carlo ground truth data. (a) Dice threshold isosurface of the ground truth absorption (gray) and reconstructions (red) for different depths. (b) 2D slices of the reconstructed absorption in ${\mathrm{mm}}^{-1}$ at z=30  mm. The true inclusion location is indicated by the blue circle.

**Fig. 17:**
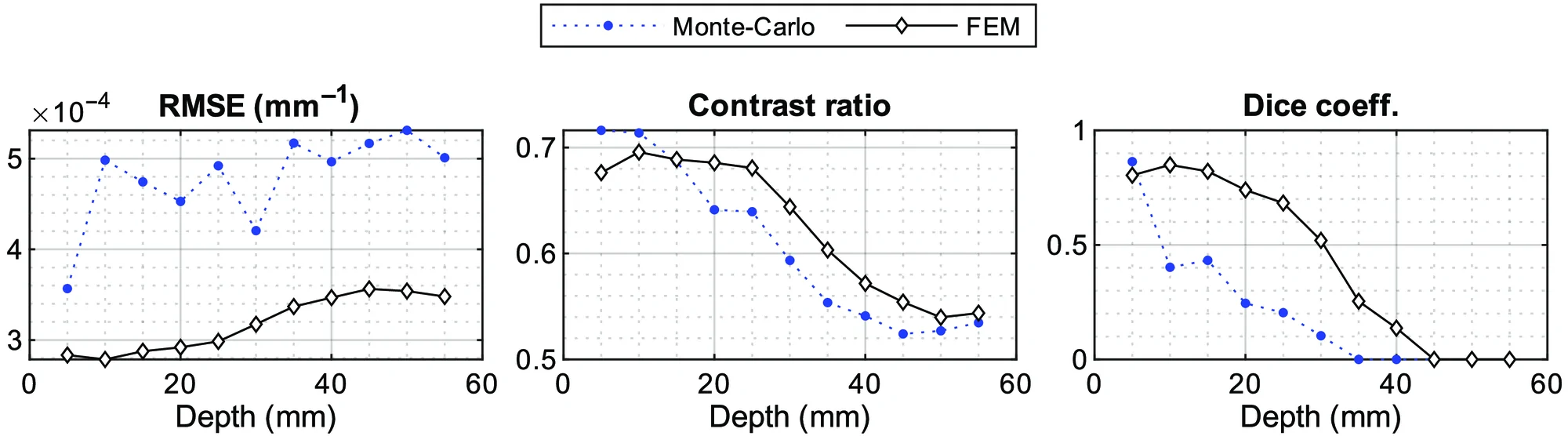
RMSE, contrast ratio, and Dice–Sørensen coefficient for absorption reconstructions from Monte Carlo data plotted against inclusion depth (top). Reconstruction timing and iteration counts (bottom). Monte Carlo ground truth reconstructions shown in dashed blue line and diffusion approximation finite element ground truth reconstructions shown in solid black with open diamonds.

Figures 16 and 17 show our model still works well with Monte Carlo data, but the depth sensitivity is limited to 30 mm. In the 3D reconstructions in Fig. 16, across all plots, there are artifacts that are reconstructed offset from the center at a depth of ∼30  mm. This region has a sensitivity more strongly associated late bins and/or large optode separation. As these data are so strongly scattered and attenuated, it is less influenced by model mismatch. These artifacts along with our good shallow inclusion reconstructions indicate that noise is our predominant source of error in the Monte Carlo reconstructions, rather than model mismatch. In general, the Monte Carlo reconstructions took longer than the reconstructions from diffusion approximation data. Both the timing and reconstruction results are in line with our results using data with synthetic noise in Sec. 4.3.3. Despite the influence of noise, our reconstructions are accurate and have good localization up to 30 mm deep.

#### 4.3.2 Complex inclusions

To assess more thoroughly the robustness of the normalization provided by the weighting matrix, reconstructions were performed using fluence data from the two complex absorption profiles: the double inclusion and the phi-shaped inclusion.

**Fig. 18:**
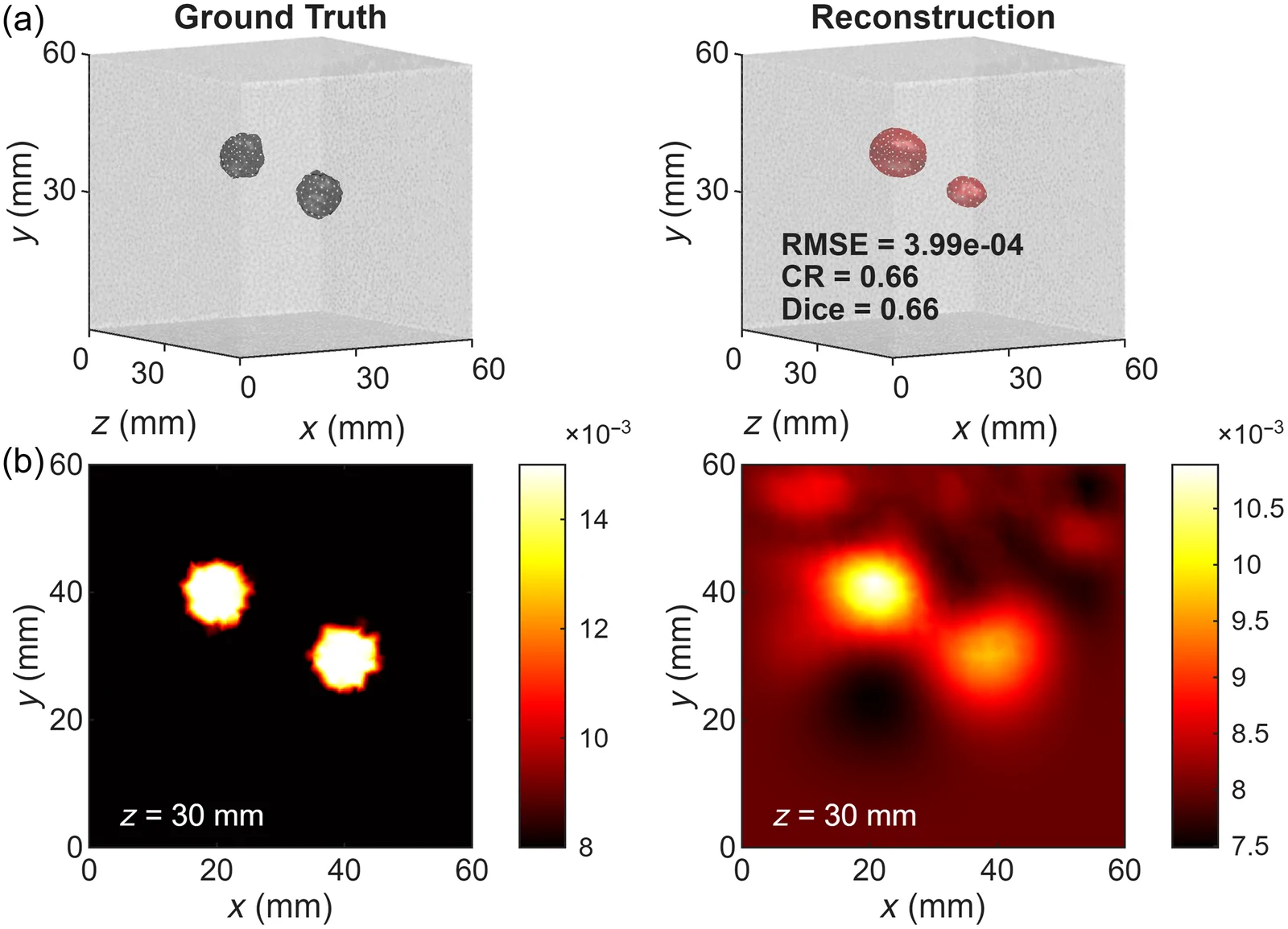
(a) Dice threshold isosurface of ground truth (gray) and weighted reconstruction for double inclusion (red). (b) 2D slice of both shown at z=30  mm with absorption in ${\mathrm{mm}}^{-1}$.

Figure 18 shows that we are able to localize both inclusions well in our reconstruction with lower contrast in the deeper inclusion, due to the lower net sensitivity in this region. It took 5 min for the reconstruction to converge, which is in line with our single inclusion reconstructions. Importantly, we use the same initial parameters as used in the single inclusion reconstructions to get these results, demonstrating the robustness of our algorithm.

**Fig. 19:**
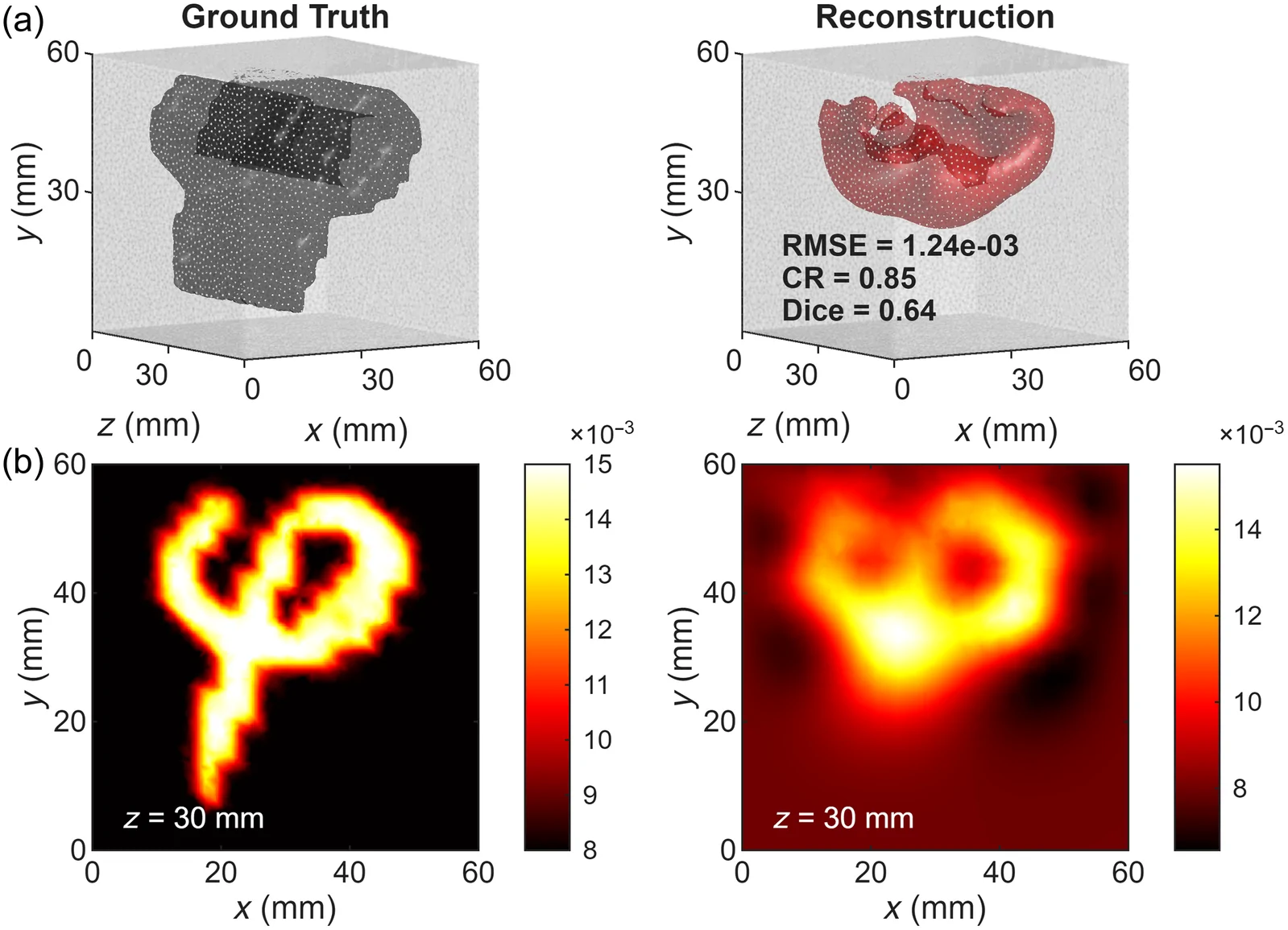
(a) Dice threshold isosurface of ground truth (gray) and weighted reconstruction for phi-shaped inclusion (red). (b) 2D slice of both shown at z=30  mm with absorption in ${\mathrm{mm}}^{-1}$.

A phi-shaped inclusion is expected to be particularly hard to reconstruct as the low absorption regions are occluded by the high absorption close to the surface. As see in Fig. 19, this is not the case, and we are able to resolve the top half of the inclusion well, including its regions of low absorption. As demonstrated in Sec. 4.2.2, our choice of basis reduces the spatial correlation of the sensitivity of our weighted measurements, enabling accurate reconstruction of this inclusion with just reflectance measurements. Beyond 40 mm, the tail of the inclusion is unable to be resolved as we begin to enter a region of lower net sensitivity. The time per iteration was in line with the single inclusion data, but it took longer to converge, so the total reconstruction took 9.2 min. This was expected as our initial guess was much further from the ground truth.

**Fig. 20:**
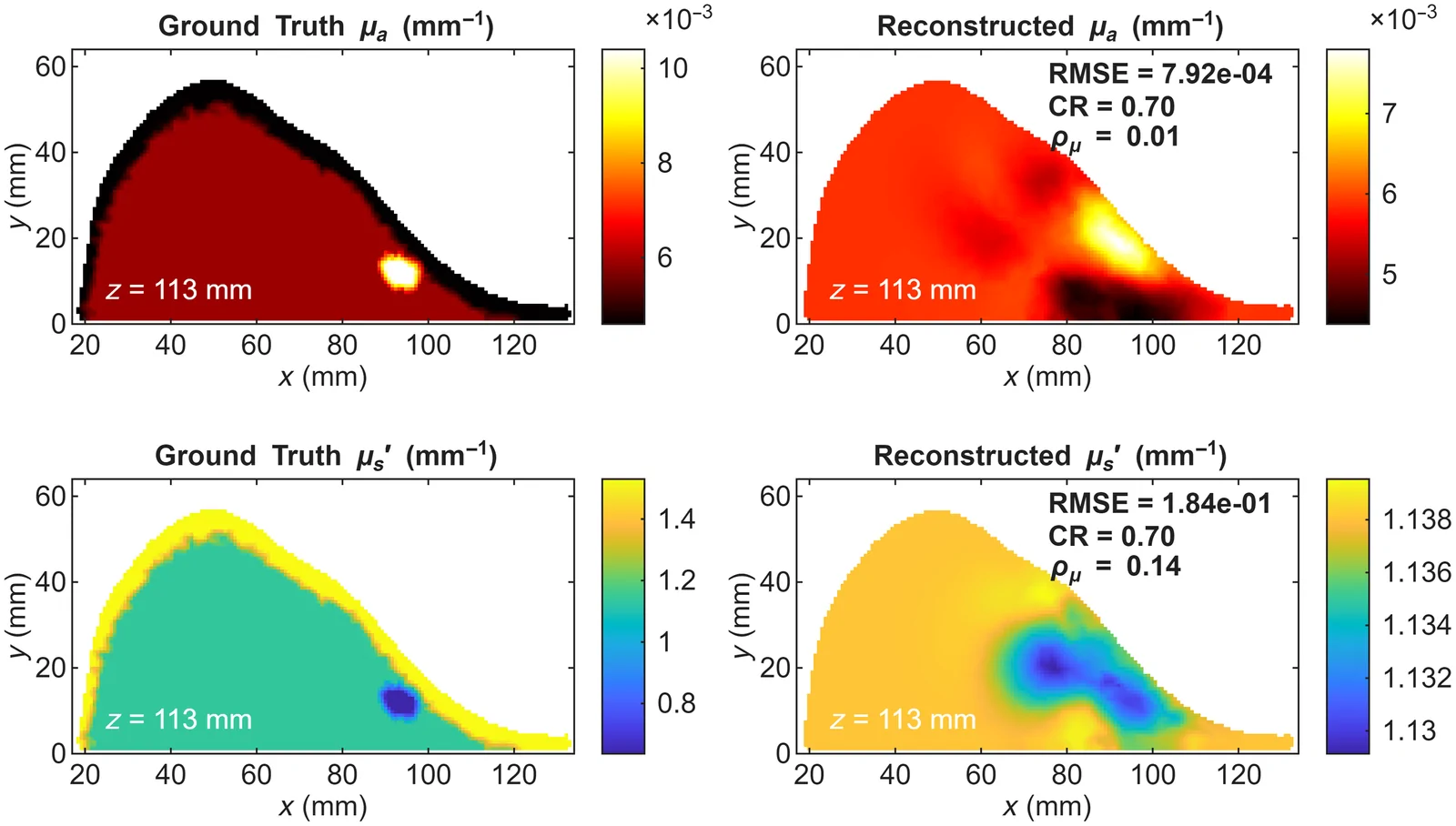
2D slices at z=133  mm of ground truth and reconstructed optical properties of the BREAST_MRI_007 digital phantom. Quoted metrics are from 3D distribution.

**Fig. 21:**
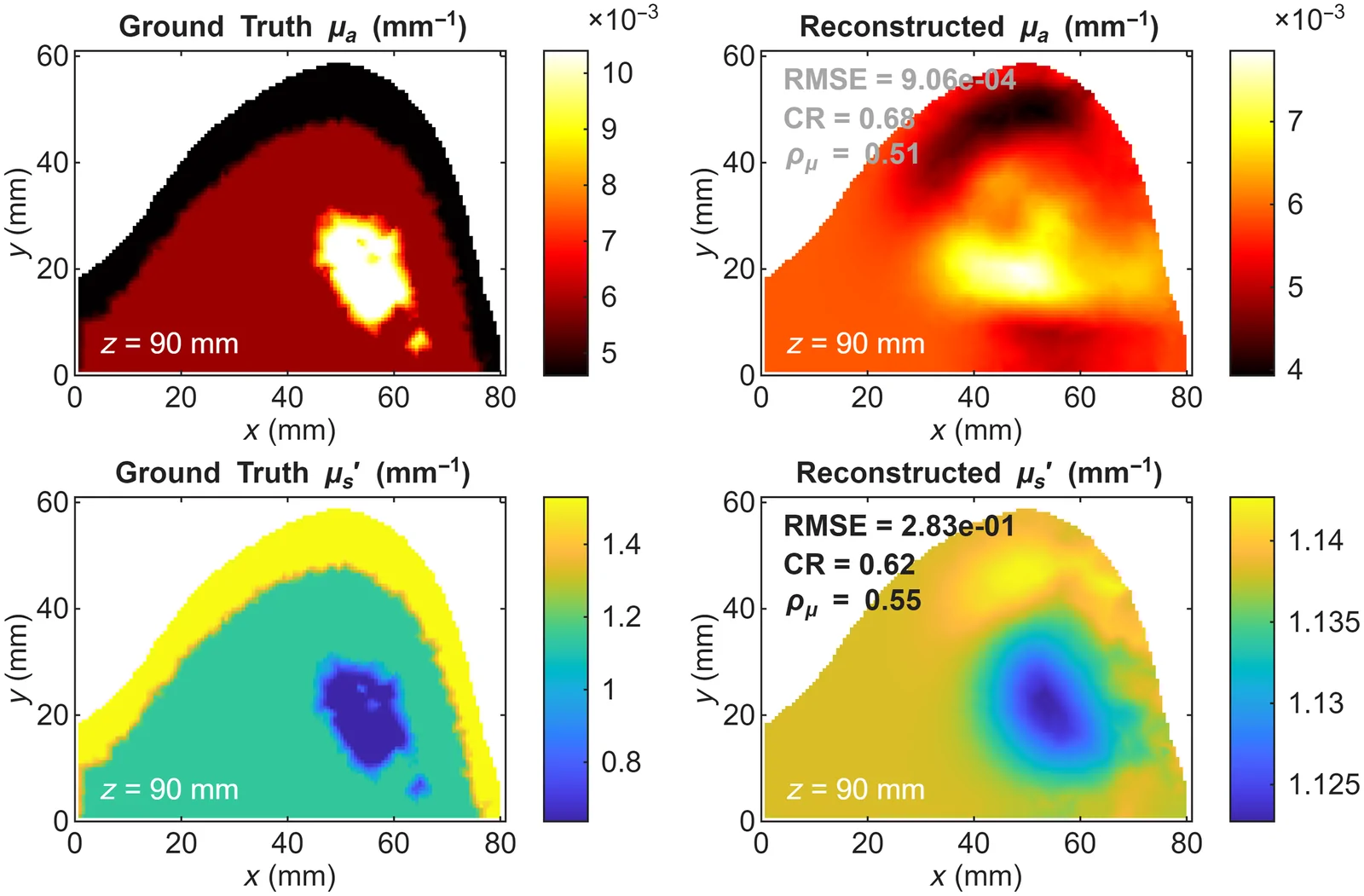
2D slices at z=90  mm of ground truth and reconstructed optical properties of the BREAST_MRI_009 digital phantom. Quoted metrics are from 3D distribution.

**Fig. 22:**
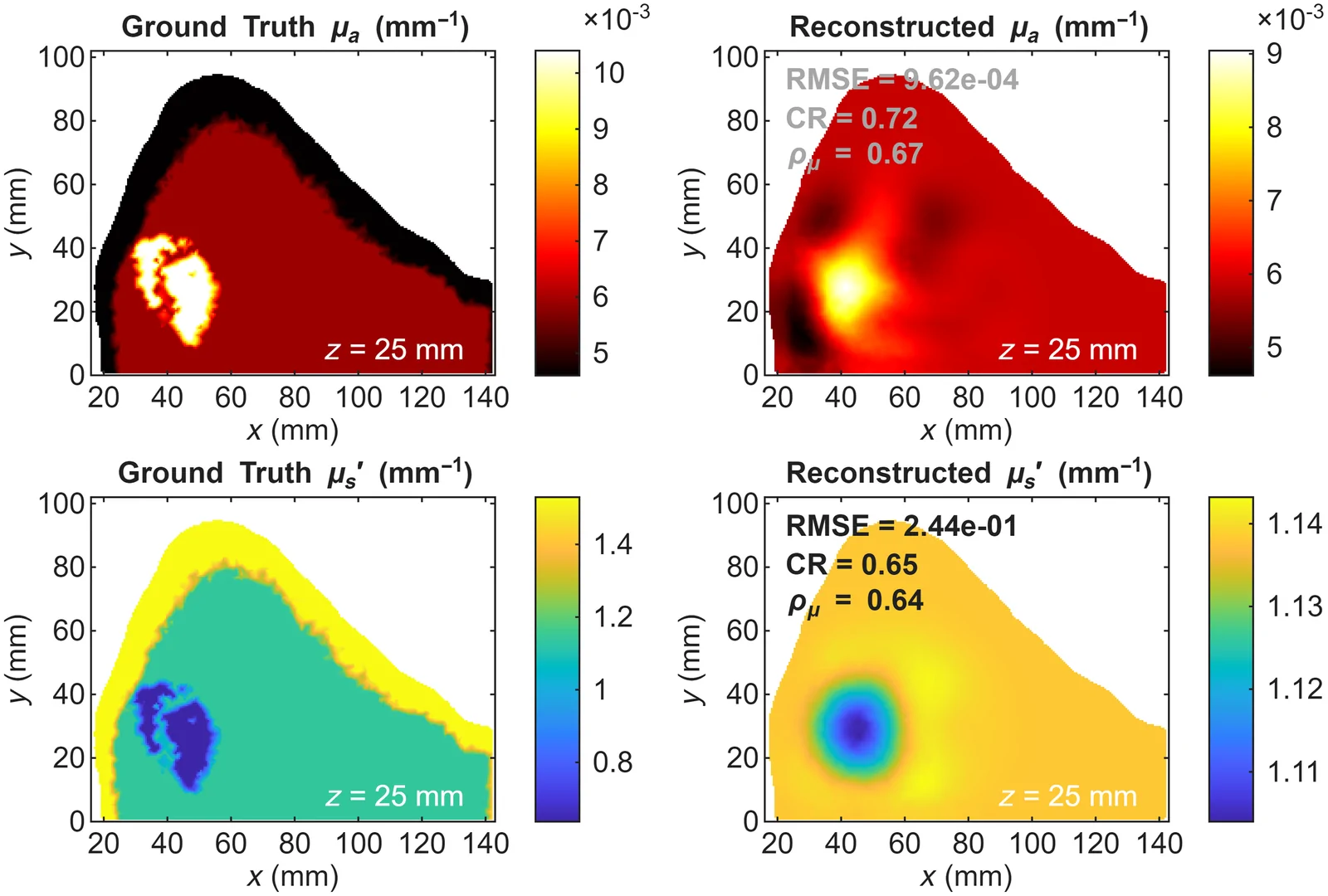
2D slices at z=25  mm of ground truth and reconstructed optical properties of the BREAST_MRI_010 digital phantom. Quoted metrics are from 3D distribution.

In realistic deployment, the optodes are unlikely to be arranged on flat plane and instead will be deformed by the surface of the target, as well as there will be heterogeneity in scattering as well as absorption. Simultaneous reconstruction of absorption and reduced scattering with realistic tissue geometry adds significant level of complexity to the problem. Despite this, Figs. 20–22 show that by defining $J_{s}$ similarly for these meshes, we do not need to retune our regularization for accurate reconstructions even with significantly different domain sizes and optode array placement or orientation.

The reconstructions in Fig. 20 have poorer localization compared with the previous results and the other breast model reconstructions as indicated by the low correlation coefficient, but we observe that the contrast ratio ratio is still very high, indicating we are still sensitive to the lesion.

In Figs. 21 and 22, we are able to localize the inclusions well despite the thick strongly scattering layer of adipose tissue as shown by the much higher correlation coefficient. Within the reconstructed ROI, we reconstruct this layer of tissue as well as the inclusion itself. Notably, by our metrics, the performance is similar to the simple binary targets despite the lower contrast in the ground truth optical properties and the additional challenge of heterogeneity in the scattering coefficient.

#### 4.3.3 Noisy ground truths

**Fig. 23:**
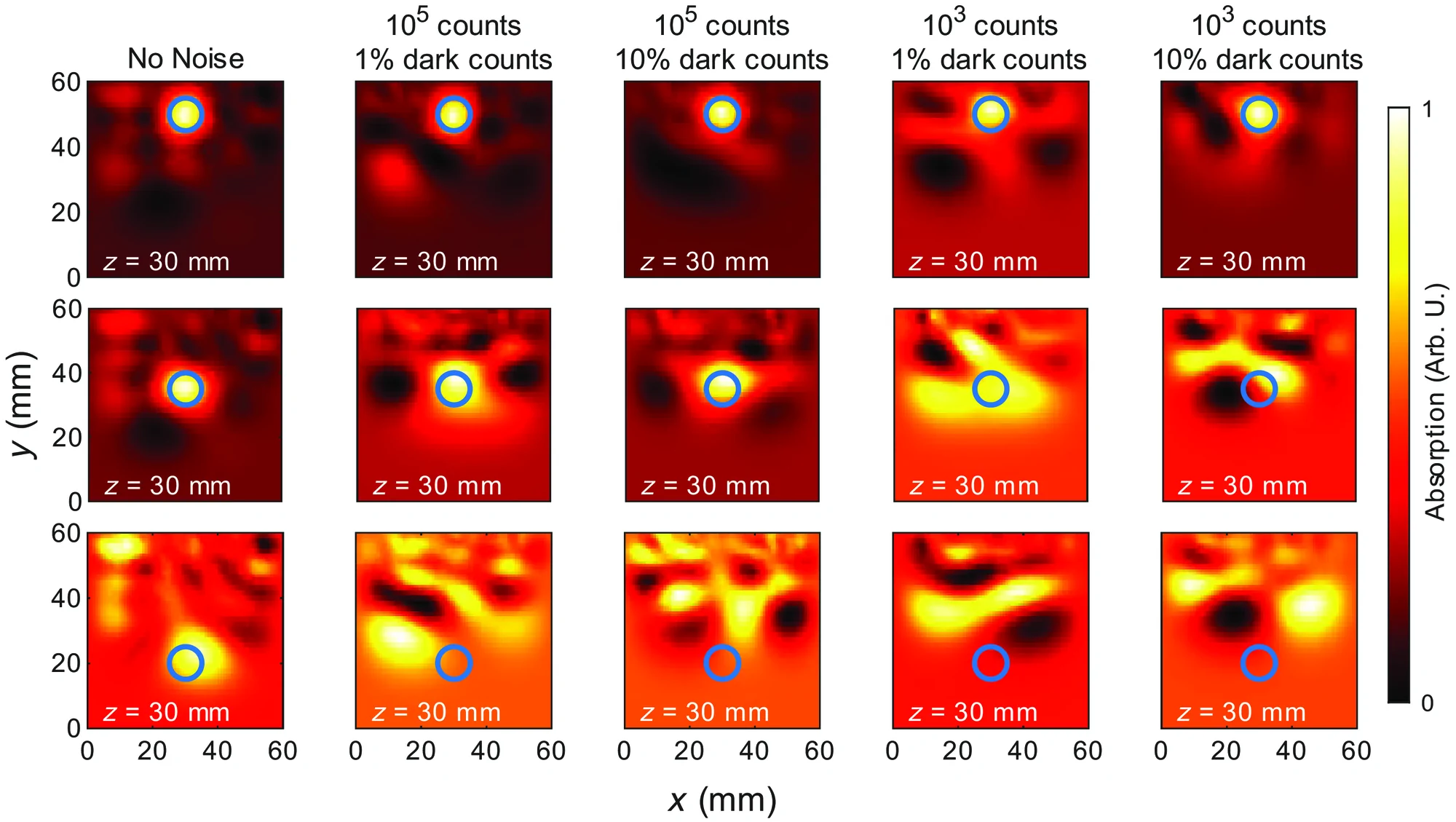
2D slice of the reconstructed absorption at z=30  mm for inclusion depths of 10, 25, and 40 mm at different levels of noise. The true inclusion location is shown as blue circles.

**Fig. 24:**
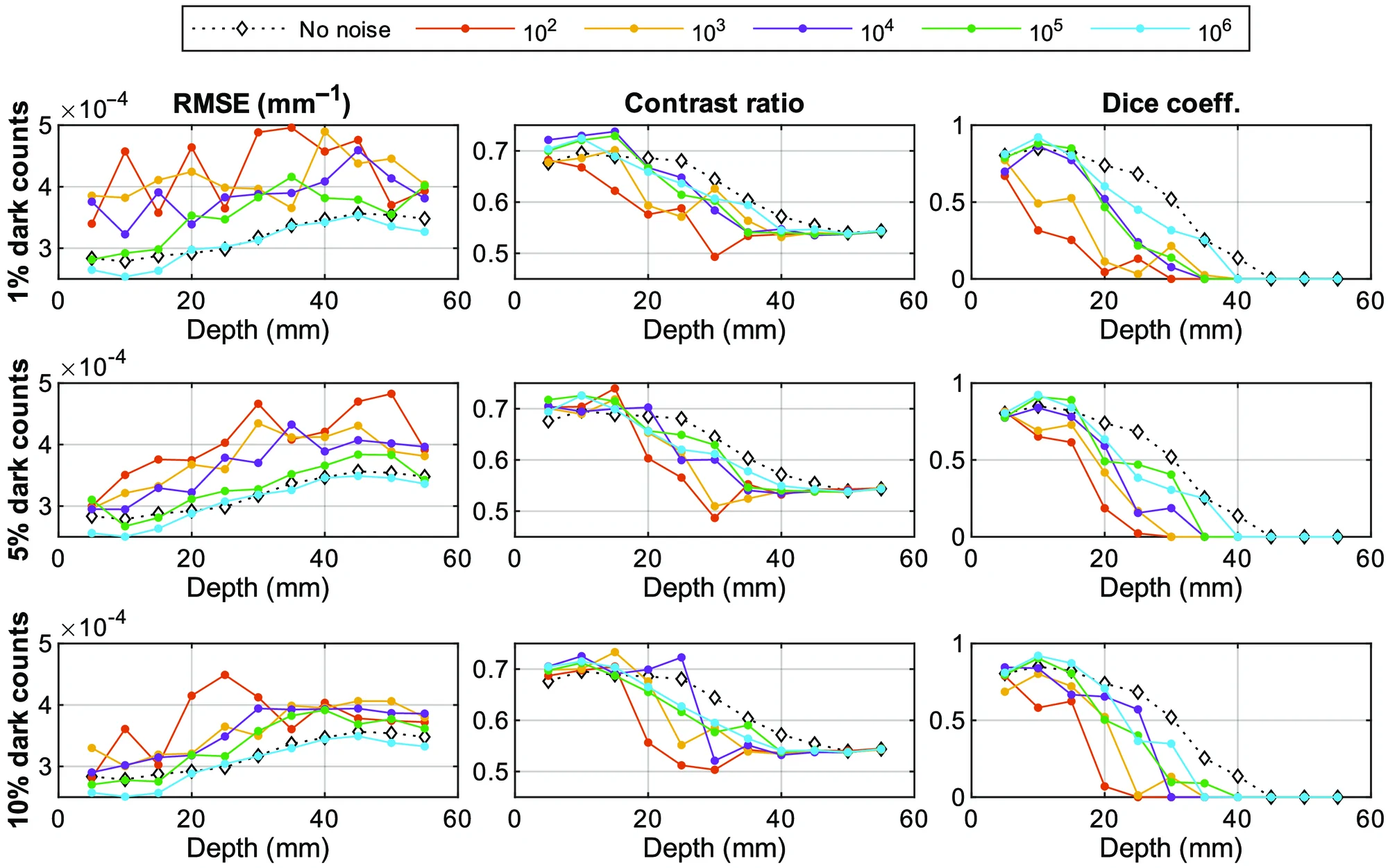
RMSE, contrast ratio, and Dice–Sørensen coefficient of absorption reconstructions for different noise levels. Each color corresponds to a measurement scaled to the indicated peak counts in the most attenuated channel with simulated shot noise. Each row shows different dark count levels of 1%, 5%, and 10% of the peak counts.

As we include different levels of noise, the quality of our reconstructions reduces as we see in Figs. 23 and 24. As discussed previously, a smaller regularizer $\lambda_{s}$ results in a higher frequency weighting, and we found $\lambda_{s}$ to be the key parameter to tune for different noise levels. For very noisy measurements, we underestimate the depth; this is in agreement with our understanding of the effect of the regularizer $\lambda_{s}$, and the reduction in the quality $J_{w}$ at deeper nodes has manifested as a reduction in the depth sensitivity of our reconstructions. Notably, the range of $\lambda_{s}$ values that worked well for each noise level (as shown in Table 1) did not change for different dark count levels. Setting a threshold for the data as described in Sec. 3.4 was enough to curtail the influence of the dark counts.

From Fig. 24, we observe a reduction in the RMSE in all our reconstructions as we increase the dark counts. Our protocol to threshold out the dark count noise floor contributes to this, but also, the offset introduced skews the noise statistics. Still, as we use less of the later gates for reconstructions with greater dark counts, the similarity and contrast for deep inclusions are worsened but are significantly less detrimental than the shot noise itself. From these simulations, we can see that as long as we are above $10^{3}$ counts in the most attenuated channel, we can resolve inclusions up to 30 mm deep with realistic noise levels.

The time per iteration with the noisy data remained around 30 s. Overall, we observed an increase in the time taken to reconstruct the scenes with noise, taking longer to converge to a solution; the reconstruction time varied between 3.5 and 8.6 min.

**Fig. 25:**
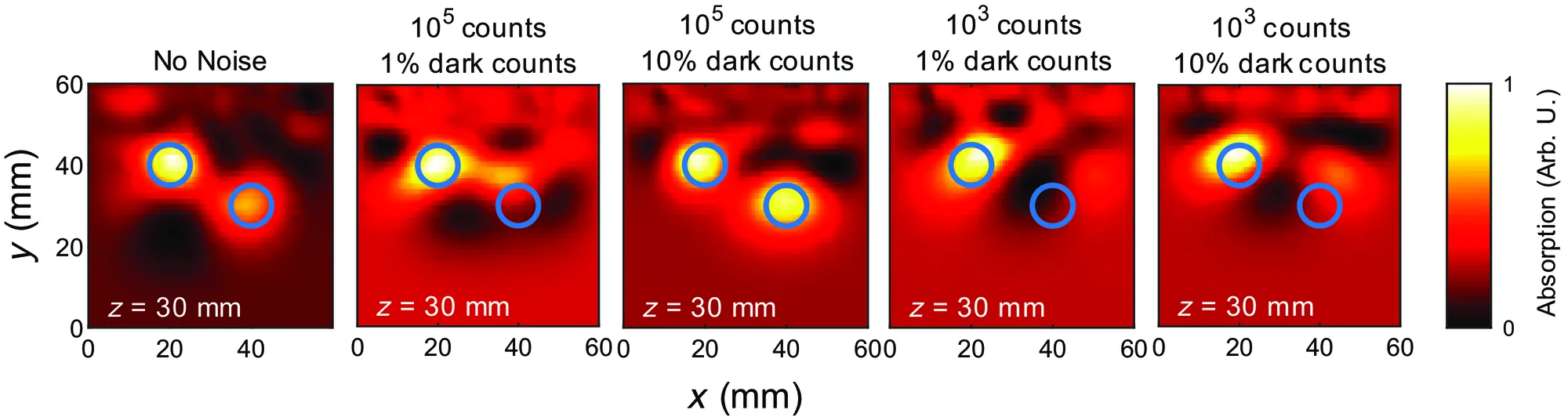
2D slice of the reconstructed absorption at z=30  mm for double inclusion at different levels of noise. The true inclusion is shown as blue circles.

**Fig. 26:**
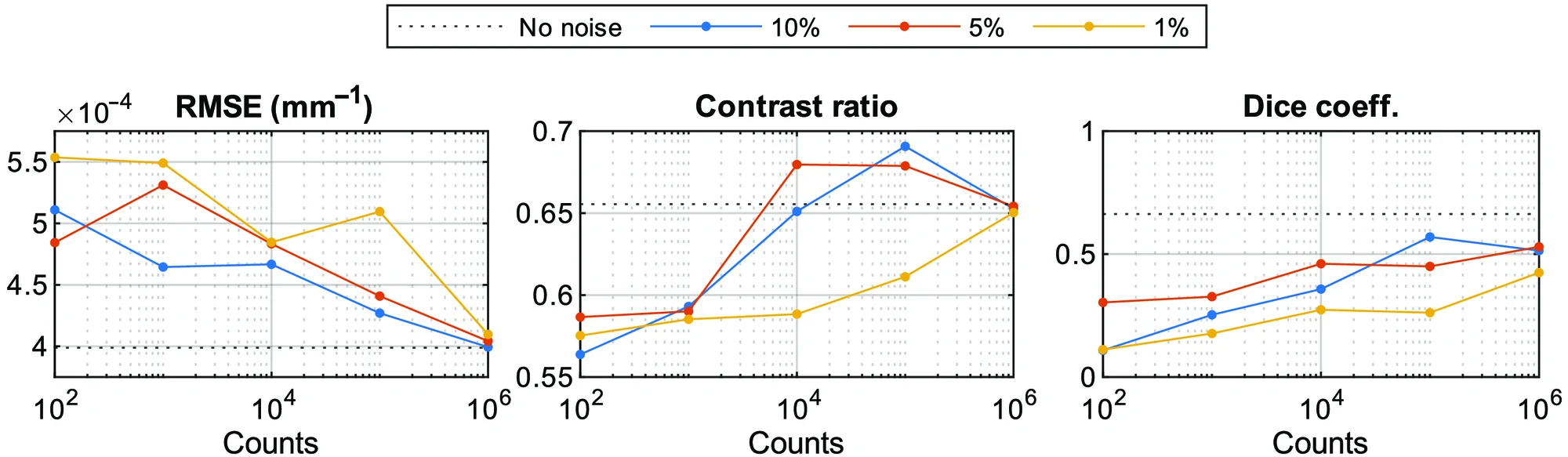
RMSE, contrast ratio, and Dice–Sørensen coefficient for absorption reconstructions of double inclusion at different shot noise levels simulated from the peak counts in the most attenuated channel as indicated on the x-axis. Color corresponds to different dark count levels.

From Figs. 25 and 26, we observe much the same trends; more noise reduces the localization accuracy and depth sensitivity. We see that for higher levels of dark counts in the measurement, our reconstructions improve as shown in both Figs. 25 and 26, and as discussed previously, we understand this as a combination of the dark count baseline skewing the noise statistics as well as our threshold protocol. In some cases, our contrast is actually better with higher dark counts, which is likely due to the more stringent threshold on later gates, which are more sensitive to noise.

The effect of noise on the timing was similar to the single inclusion case with reconstructions taking between 5.6 and 8 min.

**Fig. 27:**
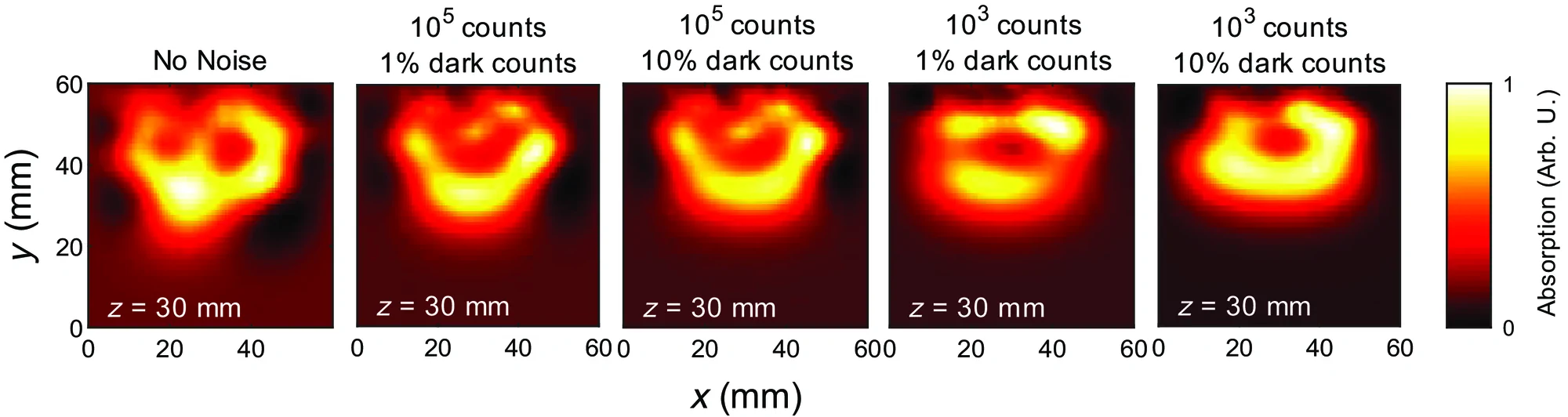
2D slice of the reconstructed absorption at z=30  mm for a phi-shaped inclusion at different levels of noise.

**Fig. 28:**
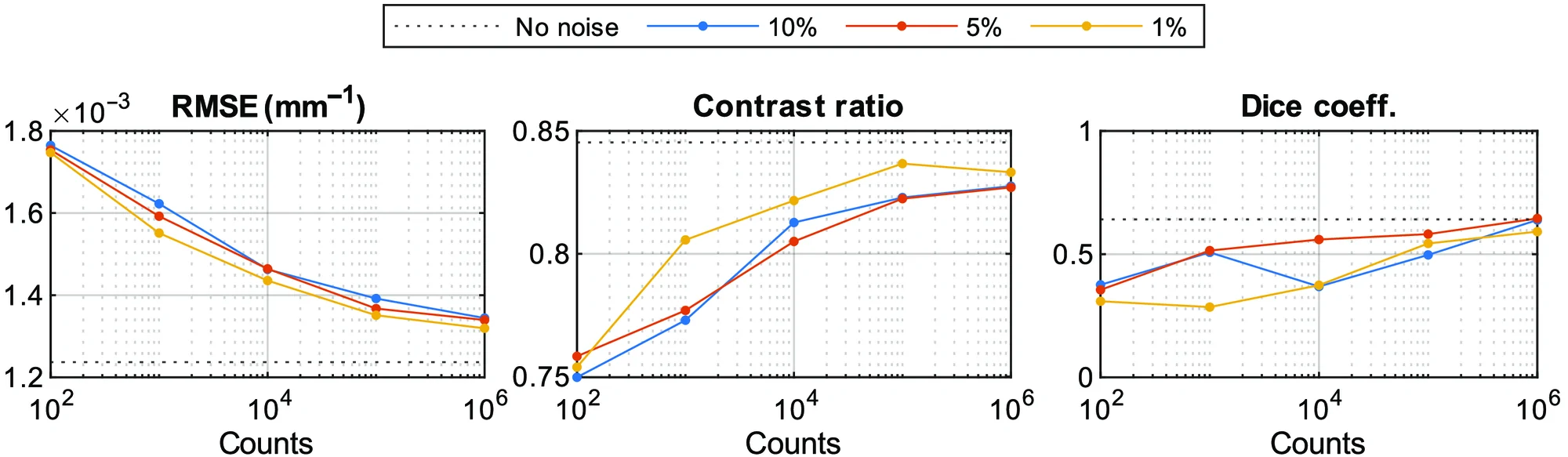
RMSE, contrast ratio, and Dice–Sørensen coefficient for absorption reconstructions of phi-shaped inclusion at different shot noise levels simulated from the peak counts in the most attenuated channel as indicated on the x-axis. Color corresponds to different dark count levels.

From Fig. 27, we directly observe the reduction in depth sensitivity with the tuning of $\lambda_{s}$. For the low count reconstruction, the span of the reconstructed inclusion is much shallower than the noise-free data. For the noisy data, as we increase the level of dark counts, we lose spatial resolution in the deeper regions. However, this is less apparent for the reconstruction with low shot noise. Overall, the reconstruction metrics in Fig. 28 indicate high-quality reconstructions for all noise levels for the phi-shaped inclusion. This is perhaps a slightly unrealistic scenario as the inclusion is very large and a cancerous lesion of this scale could be easily noticed by a clinician without a mammogram. However, it does illustrate that using our weighted scheme with a significant level of noise, we are able to resolve complex scenes with only reflectance measurements.

As previously observed, the noisy reconstruction took longer to converge, with reconstructions having a similar time per iteration but taking between 8.4 and 11.7 min to finish.

The capability of our weighted reconstruction scheme to reconstruct a variety of scenes with the same initial parameters demonstrates the efficacy of the weighting scheme as a normalization method. No individual calibration dependent on the ground truth is needed. Instead, we tune λ and $\lambda_{s}$ based on our choice of $J_{s}$ and further tune $\lambda_{s}$ based on the noise in the measurement.

## 5 Discussion

### 5.1 Sensitivity Weighting

In practical applications of DOT, completely accurate modeling of the sample boundary is difficult. Our results in Fig. 12 highlight that it is important to ignore early photon data using a scheme such as this. In a previous study, Radford et al.^61^ show that in diffuse regimes, early and late photons contribute comparably to image reconstruction, and as more late photons are used, the image quality improves. We think that it is also important to consider the limits of the model and the geometry of detection. In reflectance geometry, we need to be considerate of errors in the mesh boundary as well as the SDS. The early arriving photons in each channel interact strongly with the boundary, amplifying any error due to boundary mismatch. Late gating as shown in Fig. 12 minimizes the influence of mismatches in the sample and mesh boundary, highlighting the importance of using only late photons in the reflectance geometry. Contrary to the transmission geometry, both early and late photons are very diffuse.

We would highlight that the applications of this weighting scheme may not be limited just to DOT. There has been growing interest in using new data types for functional near-infrared spectroscopy (fNIRS).^49^^–^^52^ fNIRS is typically performed in the reflectance geometry, so the sensitivity to surface changes such as the blood–brain barrier can be overrepresented in the data. Our simulations have shown we are not limited to the familiar “banana” sensitivities typical to fNIRS and DOT, and instead, with the temporal selectivity of gated data types, we can form arbitrary (within reason) regions of sensitivity that could minimize the influence of surface measurements or amplify the sensitivity in a region of interest. Compared with picking new integral transforms or different combinations of measurands by intuition, our approach could be much more flexible. Finding the weightings is a relatively lightweight computation and can be done in advance in an application such as fNIRS. Further experimental investigation into the efficacy our weighting schemes is needed.

### 5.2 Depth and Reconstruction Quality

As discussed previously, other depth-based normalization schemes amount to just biasing the reconstruction to a certain depth, which means the extra normalization parameter is hard to ascertain without some prior information.^36^^–^^38^ For each of the noise-free reconstructions at a given inclusion depth, we do not need to tune either λ or $\lambda_{s}$ to get high-quality results; these results demonstrate that our normalization scheme is both robust and regularization parameters are weakly coupled to the ground truth.

Compared with the FEM-generated noise-free data, a larger regularizer $\lambda_{s}$ was used for the Monte Carlo data, but we did not need to tune our initial parameters for the different ground truths. This increase was expected due to the noise in the simulation. Accordingly, we found the depth sensitivity was limited to 30 mm in these cases

As we only need to tune one parameter ($\lambda_{s}$) based on the noise, we are less likely to overfit at deep nodes in the mesh and attribute noise to an inclusion. In addition, the proportionality between noise and $\lambda_{s}$ indicates the possibility to automate the choice of $\lambda_{s}$. In our implementation, we could not use this proportionality to inform our choice of $\lambda_{s}$ for the Monte Carlo reconstructions. MMC solves for Green’s function of the fluence in the mesh, so convolution of this solution with the IRF results in a modification of the counting statistics, making it hard to draw a comparison between the two datasets.

One advantage of FD data types and TD moments is that they are more robust to noise than gated data types, with higher-order moments and higher-frequency components being more sensitive to noise. When we use the weighting matrix to form wδϕ, such as these data types, the weighting amounts to an integral transform (but we also sum these transforms across different channels). Due to this, we are able to retrieve high-quality reconstructions even with very noisy data.

It is important to stipulate here that like any DOT method, the reconstruction quality is heavily dependent on the optode arrangement and, following that, the choice of $J_{s}$. Investigation of the optimum choice of both of these parameters is not trivial, and our current implementation is essentially the most intuitive best case, a bifurcated grid optode arrangement, and a scanning spot $J_{s}$. A more thoroughly defined choice of $J_{s}$ and optode arrangement could be used to improve the reconstruction quality and also reduce compute times by dimension reduction.

### 5.3 Reconstruction of Complex Inclusions

In reflectance DOT, scenes such as the double and phi-shaped inclusions are hard to resolve as they span a relatively large range of depths. Depth-based normalization techniques in this case do not work well and unnormalized schemes bias the reconstruction to shallow regions of the inclusion. In the literature, the characterization of depth sensitivity for reflectance DOT is limited to just single inclusion depth for this reason. A robust normalization scheme should provide consistent results across many depths for single inclusions as well as for complex scenes that span deep into the sample. All without tuning extra parameters based on the ground truth which, of course, is typically unknown. For all reconstructions, the complex targets, and the single inclusions, the same regularizers were used, with only $\lambda_{s}$ being tuned for different noise levels. Even still, as we can see from the quality of the reconstructions in Figs. 14, 18, and 19, $\lambda_{s}$ is independent from the ground truth, and tuning of $\lambda_{s}$ is much more intuitive than normalizing based on depth.

For the double-inclusion noisy reconstructions in Fig. 24, we observe an increase in the reconstruction quality for higher dark counts. As we noted, this is due in part to a skew in the noise statistics, but it further highlights how resilient to noise this weighting scheme is. Despite filtering out more late gate measurements for higher dark counts, we can still reconstruct deep inclusions. Taking advantage of the fact that the earlier gates will be less susceptible to noise, we can weight them differently to increase depth sensitivity.

Ideally, the reconstruction of the phi-shaped inclusion in Fig. 19 would resolve its entirety, but this result also highlights the robustness of this method. By deriving the weightings directly from Eq. (10), the depth limit is “baked-in” as regions of low sensitivity correspond to nodes where we cannot find a linear combination of measurements that are sensitive to that region. This allows us to resolve the shallower region of the inclusion without over-fitting at the deep nodes as can be the case with normalization schemes based on node depth. The dependency of spatial resolution on the dark counts in the reconstruction was much clearer in these reconstructions too. Typically, spatial resolution in DOT is associated with optode density, but we observe with these reconstructions that it is also coupled to temporal sampling, motivating the use of gated data types or bins rather than moments, especially in scenarios where device footprint is required to be compact.

The reconstructions of the breast lesion data from BREAST_MRI_009 and BREAST_MRI_010 are even more convincing than the binary absorption inclusion reconstructions. This represents a much more complex deployment of our methodology with optode placement and orientation deformed by the mesh surface, as well as a more more complex ground truth in both absorption and scattering. Despite this, with the same initial parameters and choice of $J_{s}$, we can retrieve both absorption and scattering accurately. The results from BREAST_MRI_007 are weak in terms of inclusion localization but still show high contrast in the inclusion. We would highlight here that the curvature of the surface the optodes lie on is higher than the other datasets, and the inclusion is relatively small. Even with weaker localization accuracy, the high contrast shows the reconstruction is sensitive to this inclusion, and high sensitivity to inclusions is key for diagnosis.

We think it is important here to highlight that there are many more limiting factors that will effect experimental deployment of this weighting scheme. DOT reconstructions in general are all sensitive to optode positioning errors, deformation of tissue around the optodes, and coupling variability. Detector-specific properties such as afterpulsing, timing jitter, and variable bin widths will also effect reconstruction quality. We expect very stringent calibration and data pre-processing will be required to realize this experimentally, given how much more susceptible gated data types would be to all of these effects. Even still, we think these results showcase that efficient use of rich time-domain data can enable high-quality and robust reconstructions. These reconstructions importantly have regularization parameters that are not dependent on some obfuscated combination of instrument noise, the anatomy of the target, and optode placement.

## 6 Conclusions

This work uses time-gated data types and a weighting scheme to enhance the localization of deep inclusions and reconstruct complex scenes in DOT. Motivated by improvement to SPAD array technology, we have shown by simulation that for a multiplexed time-gated detection scheme, we can take an approach inspired by transmission matrix inversion schemes in adaptive optics to derive a weighting matrix that transforms the familiar time-gated sensitivity “banana plots” of our measurements to a more favorable basis for reconstructions. With the methodology described in this study, our target basis can take any form (limited by a regularized ill-posed inversion), but our choice of basis was a Gaussian spot that scans the span of our computational phantoms. This allows us to form a measurement basis with a greatly reduced spatial correlation. We think that these localized sensitivities could be useful in fNIRS measurements and have shown by simulation that they could be used to enhance the spatial selectivity of diffuse measurements. The reduced spatial correlation among the rows of the new weighted sensitivity matrix was shown to improve the reconstruction of complex scenes and deep inclusions in reflectance DOT, a form factor we believe is critical to improve for widespread adoption of diffuse optics in clinics. This weighting scheme relies only on the computation of the time-gated sensitivity which is typically time-consuming, but we leverage the parallelizability of the FD diffusion approximation and a truncated Fourier series representation of our TD data to solve for the time-gated output completely in parallel. Applying these weightings to the residual in the objective function of our LM minimization enhances deep inclusion localization without an explicit depth prior. This is without the risk of overfitting at deep nodes or biasing the reconstruction to certain depths as is the case with other normalization schemes that do not rely on anatomical priors. No direct comparison of the reconstruction quality has been made among different depth normalization schemes, but we show good performance with our method. Critically, the choice of regularizer is weakly correlated with the ground truth.

With our simulated data, this weighted minimization scheme is shown to be able to localize absorbing inclusions up to 40 mm deep with a maximum SDS of 50 mm. In addition, we resolve more complex scenes where we have two absorbing inclusions at different depths and a phi-shaped inclusion with a low-absorption region occluded by the surrounding regions of high absorption. We deployed our algorithm on computational phantoms taken from breast cancer MRI data with heterogeneity in both absorption and scattering demonstrating a high sensitivity to simulated cancerous inclusions across a range of geometries without changing regularization parameters. For the smaller inclusion, the localization was not as accurate as the other results, but the reconstruction still showed high contrast which is important for clinical diagnosis.

To assess the experimental viability, we simulated different levels of shot noise and dark counts and found that our method was still effective and the additional regularization parameter in our method is independent of the ground truth absorption profile and only requires tuning based on the level of noise in each measurement. We found that for a shot-noise-limited detection scheme (as is typically the case in TCSPC measurements) above $10^{3}$ peak counts in the most attenuated channel, we are able to accurately resolve and locate inclusions up to 30 mm deep. As well as this, we used Monte Carlo data as a ground truth to assess if our method works with a realistic model–data mismatch. Similarly, we found the depth limit, in this case was 30 mm, but the dominant effect was noise from the statistics of the simulation rather than the model mismatch.

Given that this scheme does not rely on anatomical priors, we think it is a significant step toward low-cost DOT in clinic for point-of-care diagnoses. Although we have tried to simulate a broad range of ground truths to demonstrate the robustness of our method, verification of our methodology requires experimental deployment. In addition, the reduced reconstruction quality in one of our simulations indicates more investigation of the optode arrangement, and choice of target basis to derive the weighting matrix is needed.

## Supplementary Material

10.1117/1.JBO.31.9.097002.s01

## Data Availability

The data underlying the results presented in this paper as well as the code to generate the data and figures are available in Ref. 62, DOI: https://doi.org/10.17861/488cfea0-1215-4886-b7ea-86b4b8ea1392
