# Supplementary material for: Weighting scheme for robust depth normalization in reflectance time-domain diffuse optical tomography
Source: J Biomed Opt. 2026 Sep 15;31(9):097002. doi: 10.1117/1.JBO.31.9.097002 (PMC13576694; doi:10.1117/1.JBO.31.9.097002)
Supplement: Supplementary file 1 [file JBO_031_097002_SD001.pdf]

Supplementary Material

Reconstruction timings for different inclusions in the cubic mesh using finite element method (FEM) solution of the diffusion approximation (DA) to generate ground truth

Fig. S1 shows the total runtime, iterations to converge and time per iteration for the noise free reconstructions on the cubic mesh. Timings for the algorithm with no weighting are also shown for the 5 mm to 55 mm deep spherical inclusions.

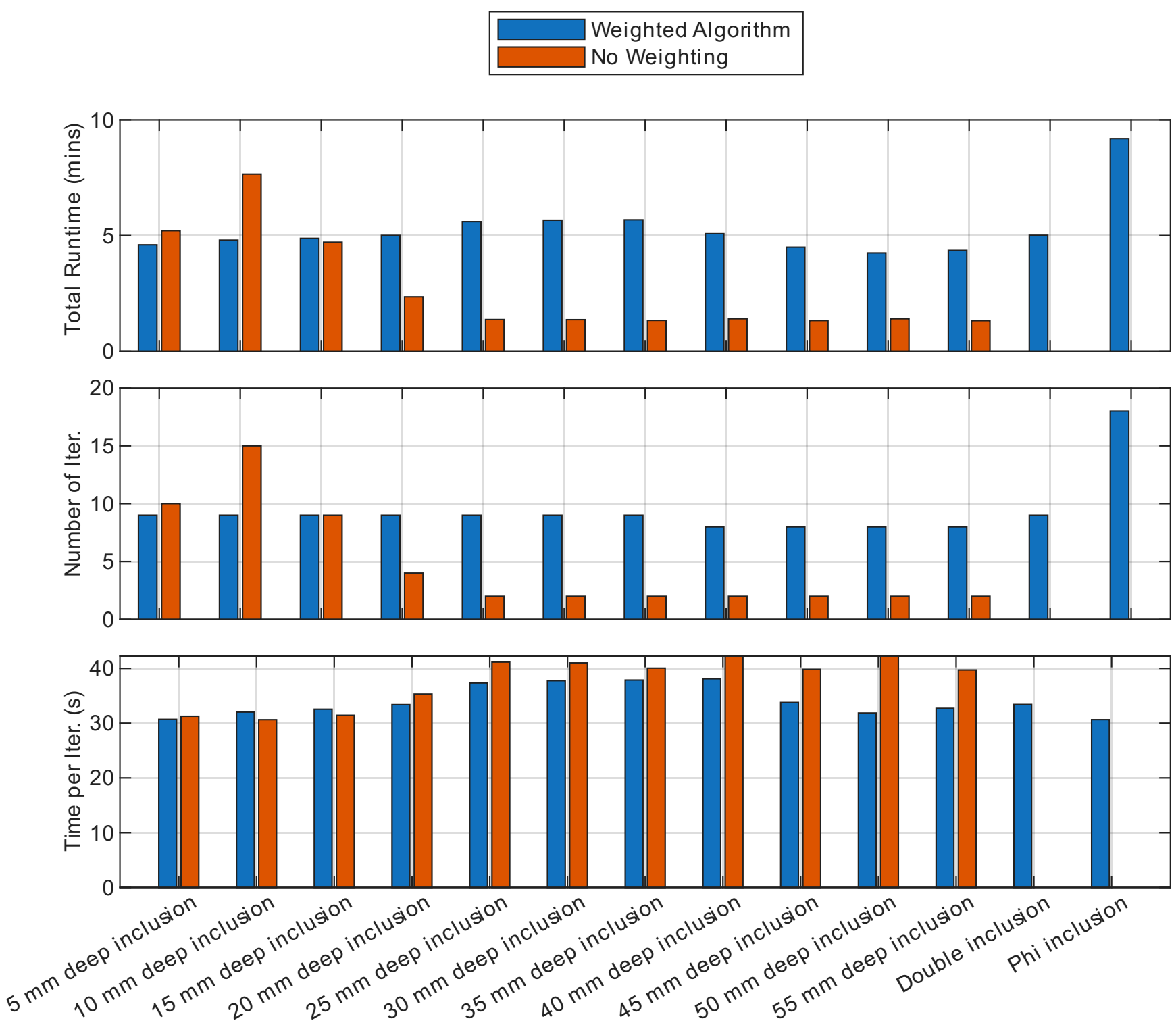

Figure S1: Runtimes and iteration counts for reconstructions in the cubic mesh

Reconstruction timings for different inclusions in the cubic mesh using Monte-Carlo ground truth data
Fig. S2 shows the total runtime, iterations to converge and time per iteration for reconstructions from with ground truth data generated using Monte-Carlo methods.

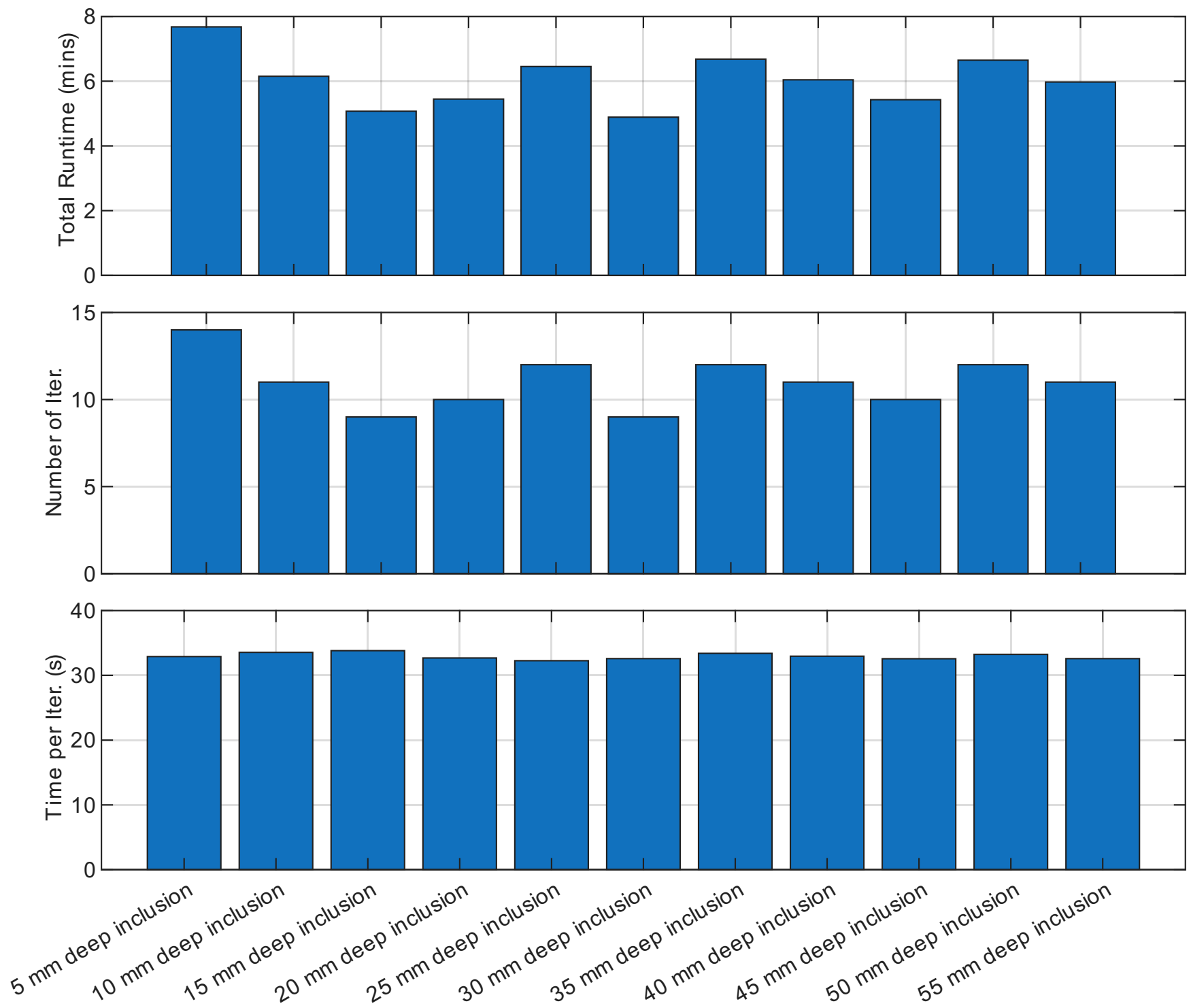

Figure S2: Runtimes and iteration counts for reconstructions in the cubic mesh

Reconstruction timings for different inclusions in the cubic mesh with noise added to the FEM/DA ground truth data

Fig. S3 shows the total runtime, iterations to converge and time per iteration for the noisy reconstructions on the cubic mesh. Similar trends in the time to converge are seen, but overall using noisy measurements increase reconstruction time.

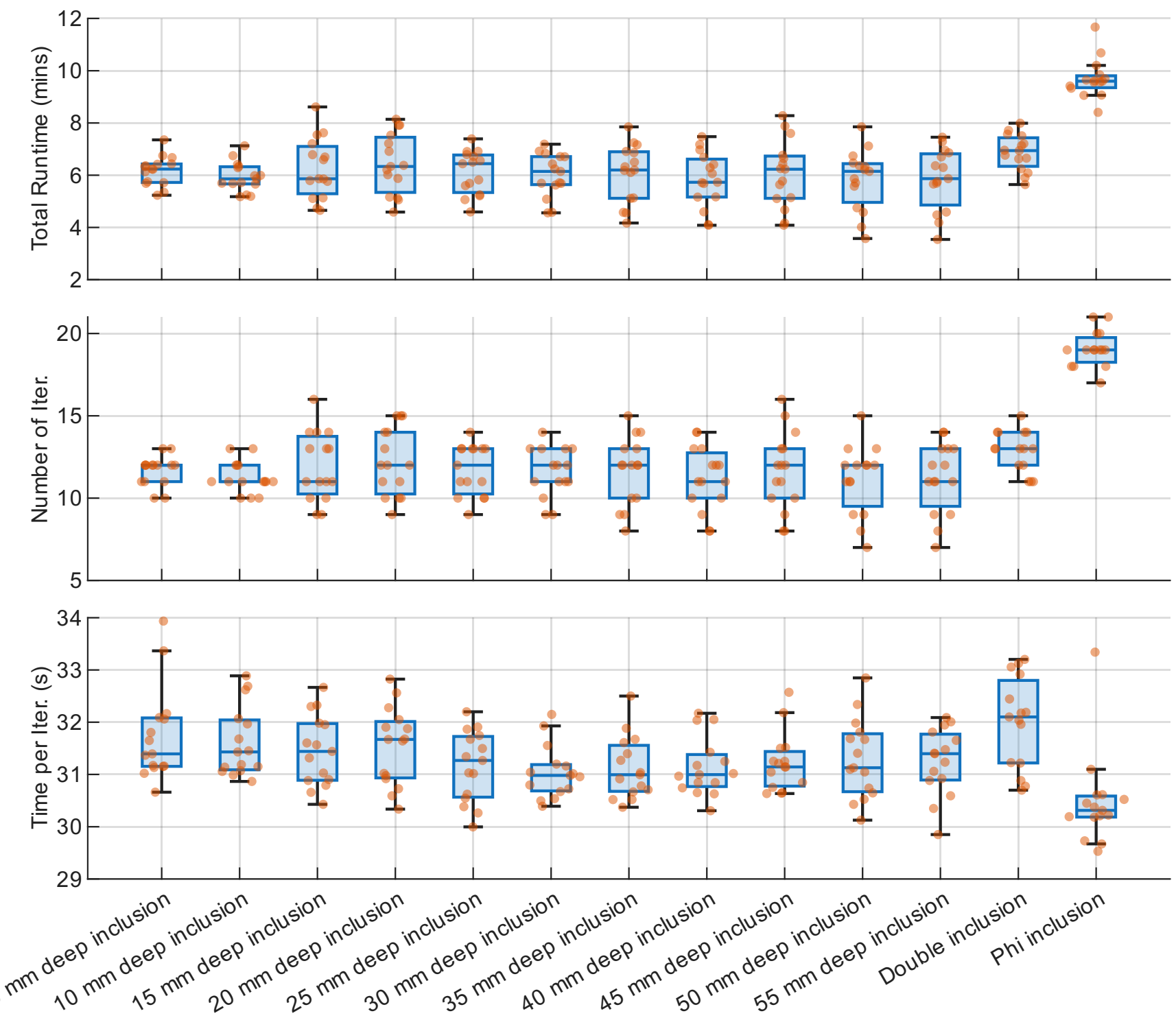

Figure S3: Runtimes and iteration counts for reconstructions in the cubic mesh with different levels of shot noise and dark counts

Reconstruction timings for breast data using FEM/DA to generate ground truth data

Adapted from three MRI datasets from Duke University[1]: BREAST_MRI_007, BREAST_MRI_009 and BREAST_MRI_010. Fig. S4 shows the total runtime, iterations to converge and time per iteration for these datasets.

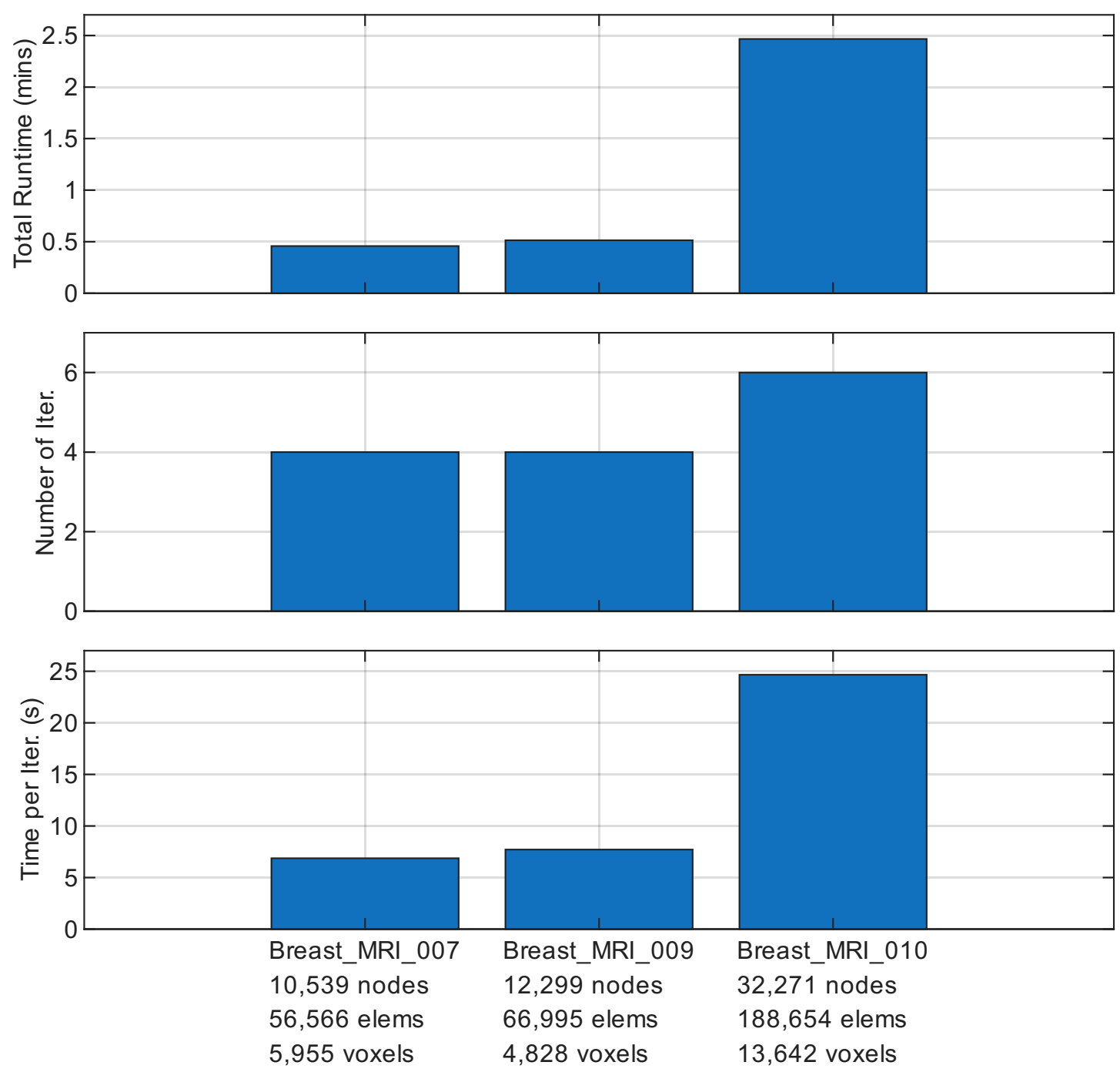

Figure S4: Runtimes and iteration counts for reconstructions of different breast meshes

References

[1] A. Saha, M. R. Harowicz, L. J. Grimm, *et al.*, "Dynamic contrast-enhanced magnetic resonance images of breast cancer patients with tumor locations," (2021).
